# Parallel Bud Mutation Sequencing Reveals that Fruit Sugar and Acid Metabolism Potentially Influence Stress in *Malus*

**DOI:** 10.3390/ijms20235988

**Published:** 2019-11-28

**Authors:** Jirong Zhao, Fei Shen, Yuan Gao, Dajiang Wang, Kun Wang

**Affiliations:** 1 Research Institute of Pomology, Chinese Academy of Agricultural Sciences/Key Laboratory of Biology and Genetic Improvement of Horticultural Crops Germplasm Resources Utilization, Ministry of Agriculture, Xingcheng 125100, China; zjr@edu.yau.cn (J.Z.); gaoyuan02@caas.cn (Y.G.); wangdajiang@caas.cn (D.W.); 2 College of Life Science, Yan’an University, Shanxi Key Lab of Chinese Jujube, Yan’an 716000, China; 3 Beijing Agro-biotechnology Research Center, Beijing Academy of Agriculture and Forestry Sciences, Beijing 100193, China; shenfei123@cau.edu.cn

**Keywords:** RNA-seq, resequencing, co-expression module, *Malus domestica* Borkh, sugar, acid, plant stress, fruit development

## Abstract

Apple sugar and acid are the most important traits of apple fruit. Bud sport cultivars can provide abundant research materials for functional gene studies in apple. In this study, using bud sport materials with a rather different sugar and acid flavor, i.e., “Jonathan” and “Sweet Jonathan”, we profiled the whole genome variations and transcriptional regulatory network during fruit developmental stages using whole genome sequencing and RNA-sequencing. Variation analysis identified 4,198,955 SNPs, 319,494 InDels, and 32,434 SVs between the two cultivars. In total, 4313 differentially expressed genes among all of the d 44,399 genes expressed were identified between the two cultivars during fruit development, and functional analysis revealed stress response and signal transduction related genes were enriched. Using 24,047 genes with a more variable expression value, we constructed 28 co-expression modules by weighted correlation network analysis. Deciphering of 14 co-expression modules associated with sugar or acid accumulation during fruit development revealed the hub genes associated with sugar and acid metabolism, e.g., *Md*DSP4, *Md*INVE, and *Md*STP7. Furthermore, exploration of the intra network of the co-expression module indicated the close relationship between sugar and acid metabolism or sugar and stress. Motif-based sequence analysis of the 17 differentially expressed ATP-binding cassette transporter genes and Yeast one-hybrid assay identified and confirmed a transcription factor, *Md*BPC6, regulating the ATP-binding cassette (ABC) transporter genes and potentially participating in the apple fruit development or stress response. Collectively, all of the results demonstrated the use of parallel bud mutation sequencing and identified hub genes, and inferred regulatory relationships providing new information about apple fruit sugar and acid accumulation or stress response.

## 1. Introduction

Apple (*Malus domestica*) is one of the most popular and important fruit trees grown worldwide. Bud mutation is important for apple breeding. Out of 143 new cultivars of apple with known parentage, marketed in the USA and Canada from1942 to 1952, nearly 25% originated as bud sports [1]. In China, the cultivar “Fuji” and its bud sport cultivars account for more than 60% of production [2]. Many bud sports are identifiable phenotypically by fruit color and shape, ripening period, branching habit, etc.

Bud sport cultivars provide abundant research materials for functional gene studies. Next generation sequencing (NGS), including whole genome sequencing and RNA-sequencing (RNA-seq), have been widely used to understand both the genetic variation and gene expression differences between sport cultivars. El-Sharkawy et al. conducted transcriptome analysis of an apple yellow fruit somatic mutation and identified a gene network module highly associated with anthocyanin and epigenetic regulation [3]. Guo et al. performed comparative RNA-seq profiling of berry development between table grape “Kyoho” and its early-ripening mutant “Fengzao” to decipher the genes contributing to the early ripening [4]. Watson et al. confirmed that somatic mutation accumulation was independent of vegetative life span in *Arabidopsis* by genomic sequencing of progeny from young and old plants [5]. Zhang et al. investigated heritable genomic variation induced by tissue culture in rice by whole genome resequencing of an extensively selfed somaclonal line and its wild type [6].

Both metabolism and accumulation of sugar and acids in apple are developmental stage-dependent activities [7]. Apple fruit acidity is primarily controlled by the major gene or QTL on chromosome 16, called malic acid (Ma) alongside a significant QTL on chromosome 8 and a few others. Jia et al. reported three hierarchical epistatic genes (*Md*SAUR37, *Md*PP2CH, and *Md*ALMTII) controlled apple fruit acidity [8]. An Ma10 gene encoding P-type ATPase is also involved in fruit organic acid accumulation in apple [9]. Bai et al. reported that a set of intramodular hub genes can play a role in regulating Ma1 via co-expression analysis across different apple cultivars [10]. *MdMYB1* also regulates anthocyanin and malate accumulation by directly facilitating their transport into vacuoles in apples [11].

Apple is unique in its metabolism and accumulation of sugars [12]. More than 80% of the total carbon flux is through fructose [7]. The characteristics of sugar transporters may differ between apple and other plants [13]. Roles in unloading and changes in expression during fruit development have been preliminarily reported for *Md*SOT and *Md*SUT1 [14,15]. Li et al. identified some members of gene families encoding transporters (including *Md*SUT, *Md*TMT, and *Md*vGT) and analyzed the relationship of their transcripts with sugar accumulation during fruit development of “Greensleeves” apple [7]. Wei et al. identified *SUT*, *MST*, and *SWEET* genes in the apple genome through phylogenetic analysis and compared them primarily with *Arabidopsis* transporters [13].

Plant sugars may also act as signals that modulate plant stress responses [16]. Sorbitol modulates resistance to *Alternaria alternata* by regulating the expression of an NLR resistance gene in apple [17]. Wu et al. reported that suppressing sorbitol synthesis substantially altered the global expression profile of stress response genes in apple, especially disease resistance genes [12]. Apple sucrose transporter *Md*SUT2.2 is a phosphorylation target for protein kinase *Md*CIPK22 in response to drought [9].

In this study, two parallel cultivars with quite different sugar and acid flavor, i.e., “Jonathan” and its bud sports cultivar “Sweet Jonathan” were studied. The dynamic change in the flavor content in Jonathan and Sweet Jonathan was measured during fruit development. The genetic base and expression feature of key genes involved in sugar and acid mechanism were explored by whole genome sequencing and RNA-seq technology.

## 2. Results

### 2.1. Distinct Components of Sugar and Acid Between Jonathan and Sweet Jonathan during Fruit Development

**Figure 1:**
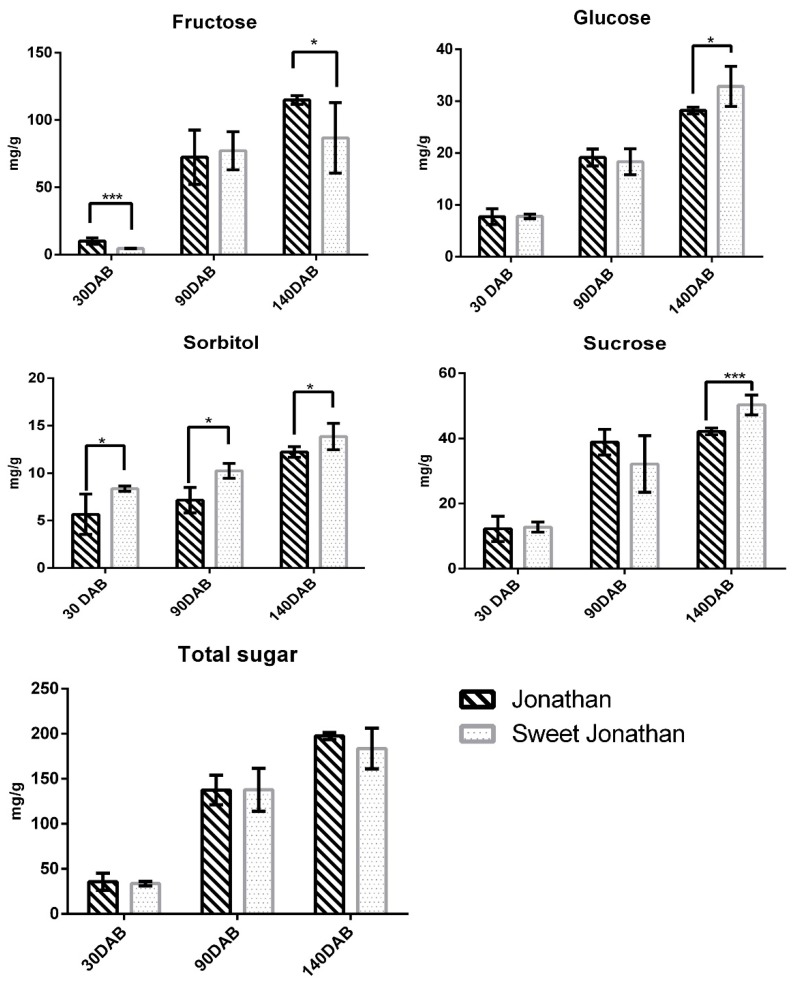
Content of individual sugar and sorbitol in apple fruit 30 days after bloom (30 DAB), 90 days (90 DAB) after bloom (90 DAB), and 140 days after bloom (140 DAB). * *p*-value < 0.05, *** *p*-value < 0.001. The error bar represents the standard deviation (SD) among the replicates.

**Figure 2:**
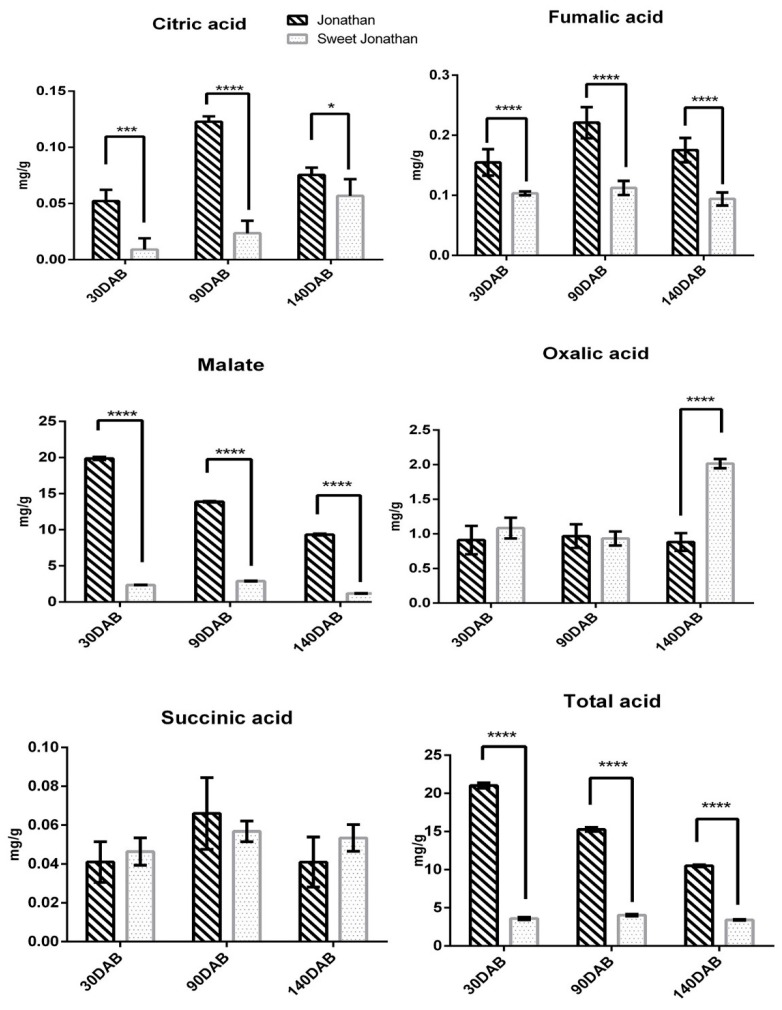
Content of acid in apple fruit 30 days after bloom (30 DAB), 90 days (90 DAB) after bloom, and 140 days after bloom (140 DAB). * *p*-value < 0.05, *** *p*-value < 0.001, **** *p*-value < 0.0001. The error bar represents the standard deviation (SD) among the replicates.

Apple fruits with five biological replicates from two parallel cultivars, Jonathan and its bud mutant cultivar Sweet Jonathan, were collected for the investigation of variations in the sugar and acid concentrations in apple fruit at juvenile, expanding, and mature stages. As the fruits developed from juvenile to mature stages, sorbitol, glucose, fructose, sucrose, and total soluble sugar per unit fresh weight increased continuously. The sorbitol content of Jonathan was significantly higher than that of Sweet Jonathan across fruit development (*p* < 0.05), however, there was no significant difference in the total sugar content between Jonathan and Sweet Jonathan during the other period (Figure 1). The total organic acid content of Jonathan was higher than that of Sweet Jonathan in various stages of fruit development. The content of organic acid of Jonathan decreased significantly with fruit development, while that of Sweet Jonathan was almost unchanged. Malic acid was the main component of the organic acid composition of Jonathan and Sweet Jonathan. In each period, the content of malic acid and fumaric acid in Jonathan was significantly higher than that of Sweet Jonathan (*p* < 0.05) (Figure 2).

### 2.2. Whole Genome Sequencing of Jonathan and Sweet Jonathan Enables Identification of Extensive Genetic Mutations between Jonathan and Sweet Jonathan

In order to profile the variations between Jonathan and Sweet Jonathan across genome, whole genome resequencing was performed. A total of 342,633,631 and 364,779,402 filtered reads were obtained for Jonathan and Sweet Jonathan, respectively. The sequencing reads provided on average 50× coverage for the apple DH genome. Approximately 69.27 M reads were mapped to the apple DH genome and approximately 60 M reads were uniquely mapped reads (Table S2).

**Figure 3:**
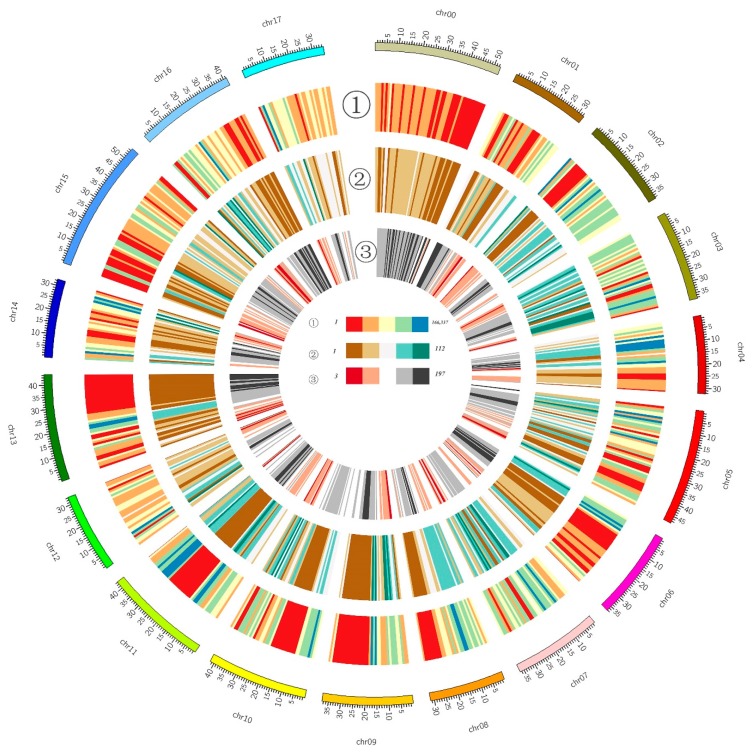
Circular overview of the variations between Jonathan and Sweet Jonathan. The outer circle, number of SNVs between Jonathan and Sweet Jonathan within a 1 M bp window; the intermediate circle, number of SVs between Jonathan and Sweet Jonathan within a 1 M bp; and the inner circle, numbers of genes within a 1 M bp window.

After variation analysis, a total of 4,198,955 SNPs and 319,494 InDels were identified between Jonathan and Sweet Jonathan (Table S3). The distribution of single nucleotide variants (SNVs) was not quite even across the genome (Figure 3 and Table S3). There was no significant correlation (*p* > 0.05) between the length of chromosomes and SNPs, InDels or SNVs, which is different from previous reports (Table S3). Functional annotation of SNVs suggested that more than 56% InDels and 68% SNPs were distributed in intergenic regions, whereas 2% InDels and 5% SNPs were annotated in exonic regions, and 1% InDels and 8% SNPs were located in upstream regions (or promoter regions) of genes (Table S4). A total of 5487 destructive mutations (frame shift InDels, stop gain and stop loss) were identified in the coding sequence (CDS) of 3,872 genes (Table S5).

In addition, large segment structure variations (SVs) were also detected and the genotypes of the two cultivars were compared with each other. A total of 32,434 different types of SVs (deletion, insertion, duplication, translocation, and inversion) were detected (Table S6). Among them, large segment deletions and insertions occupied more than 30,347 (93%), of which 1,100 were located in the exonic region of 1,035 genes, and 3,309 were located in the upstream regions of 3,701 genes (Table S7).

### 2.3. The Transcriptome Profiles of Jonatha’ and Sweet Jonathan during Fruit Development

**Figure 4:**
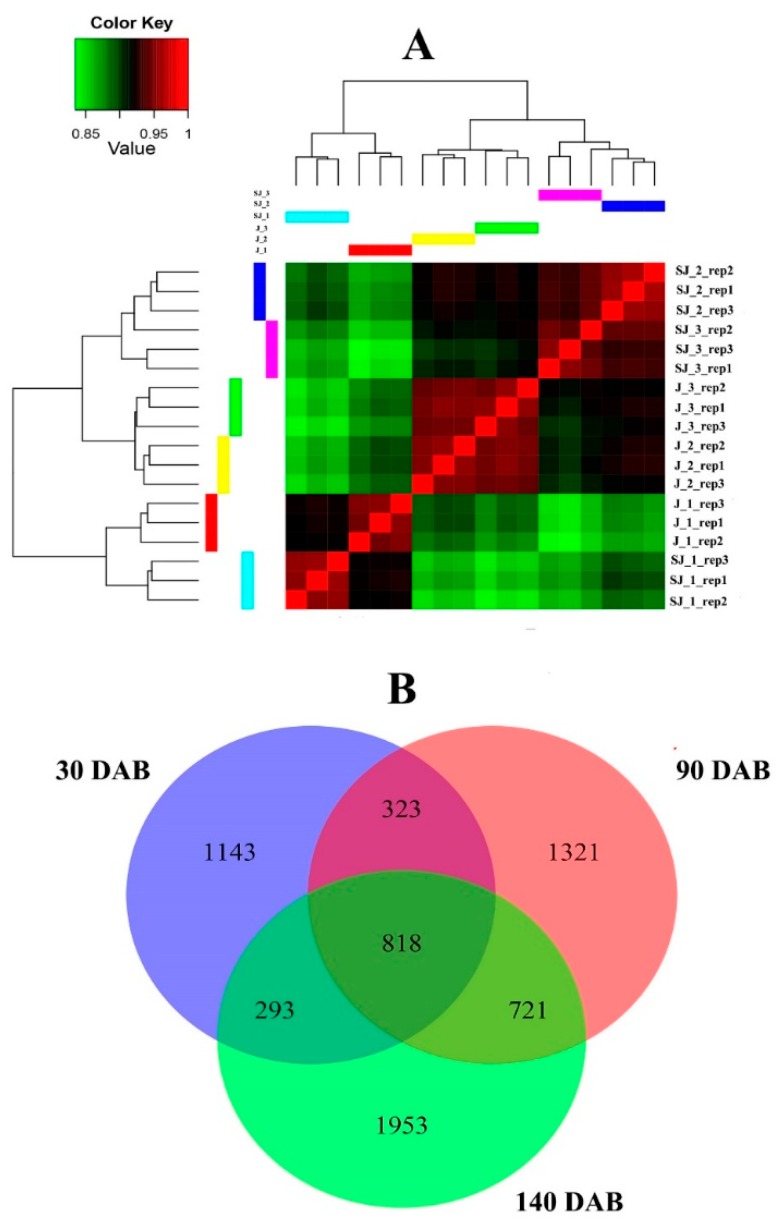
Hierarchical cluster analysis (**A**) based on the gene expression of all the samples and Venn diagram of differentially expressed genes at three stages between Jonathan and Sweet Jonathan (**B**). “J” (or “SJ”) represent the samples from Jonathan (or Sweet Jonathan) and the following number represents the different stages, 30, 90, and 140 days after bloom (30/90/140DAB).

RNA-seq was preformed to detect the transcriptome profile differences of Jonathan and Sweet Jonathan, during three fruit developmental stages (juvenile, expanding, and mature stage). Eighteen RNA-seq libraries from three biological replicates of Jonathan and Sweet Jonathan sampled at the three stages were constructed, followed by high-throughput sequencing. After removing low-quality reads and those derived from rRNA, 244.82 M total reads input for mapping, of which 87.7% were aligned to the apple DH genome (Table S8). Overall, 90,265 transcripts from 52,854 genes (46,558 referenced and 6294 nonreferenced genes) were assembled and 15,839 multi-transcript loci (~1.7 transcripts per locus) were identified (Table S9). All genes were quantified and assign with the fragments per kilobase of transcript per million mapped (FPKM) reads; 44,399 genes were detected to be expressed across three fruit developmental stages. On average, more than 31,129 (~58%) genes had low expression levels (FPKM < 1), and only 7895 (~15%) genes had high expression levels (FPKM > 10) (Table S10). The FPKM values of three representative genes, MD05G1236300, MD05G1261100, and MD08G1128800 were closely correlated with their relative expressions based on RT-qPCR (R^2^ = 0.98–0.99, *p* < 0.01) (Figure S1). The hierarchical cluster analysis revealed a good correlation between biological replicates in both Jonathan and Sweet Jonathan (Figure 4).

In total, 4313 differentially expressed genes (DEGs) were detected between Jonathan and Sweet Jonathan across different fruit developmental stages (Figure 4 and Table S11). Gene ontology (GO) enrichment analysis of DEGs revealed that 27 GO terms were enriched in at least one developmental stage, in particular, stress- and signaling-related GO terms were enriched across all fruit development stages (Tables S12–S14). Similarly, Kyoto Encyclopedia of Genes and Genomes (KEGG) metabolism pathway analysis revealed that DEGs were distributed among 119 pathway terms across the different stages (Table S15).

### 2.4. Identification of Co-Expression Modules

**Figure 5:**
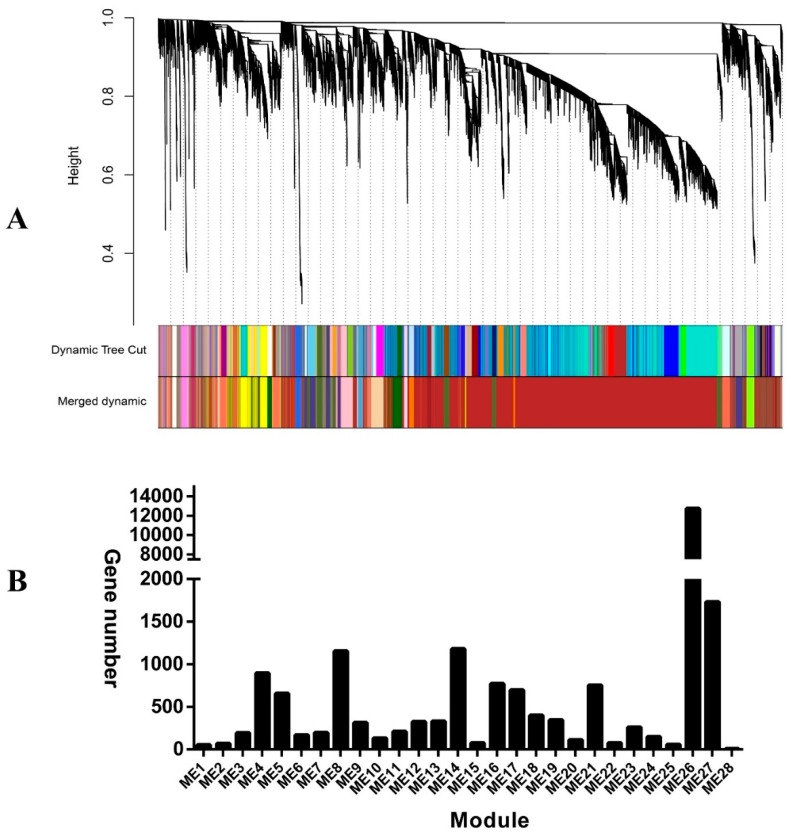
Clustering dendrogram of genes (**A**), with dissimilarity based on topological overlap, together with assigned module colors. Gene number harbored in each module (**B**).

**Figure 6:**
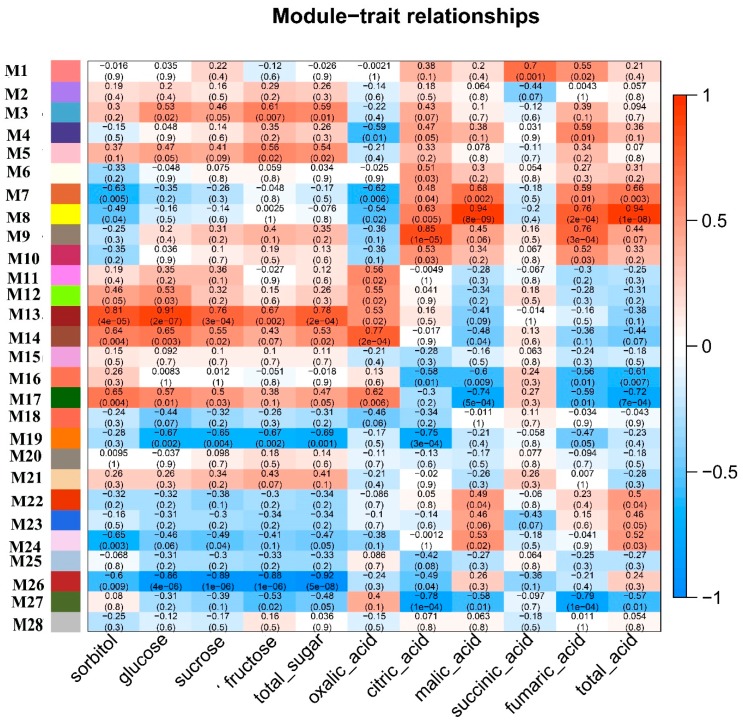
Module-trait associations. Each row corresponds to a module eigengene, and each column to a trait. Each cell contains the corresponding correlation and *P*-value. The table is color coded by correlation according to the color legend.

In order to decipher the internal regulation network, we performed weighted co-expression gene network analysis based on the expression dataset [18,19]. After filtering based on the expression and variation across all the samples, the 24,047 remaining genes fell into 28 co-expression modules (M1 to M28), and the number of genes harbored in co-expression modules ranged from nine to 12,732 (Figure 5). We related modules to sugar and acid traits to identify important hub genes. 14 co-expression modules were identified to be related to at least one sugar and acid trait during the fruit developmental stages. Among them, M13 and M26 were significantly related with all measured sugar traits (sorbitol, glucose, fructose, sucrose, and total sugar) (*p*-value < 0.01), and M19 was significantly associated with glucose, fructose, sucrose, and total sugar (*p*-value < 0.01) (Figure 6 and Table S17). Compared with other sugar and acid traits, sorbitol was significantly related to more co-expression modules (six) (*p*-value < 0.01) (Figure 6 and Table S16). As the major organic acid composition, four modules (M7, M8, M16, and M17) were detected to be significantly associated with the malate content (Figure 6 and Table S16). We also detected the following three substance-specific modules: M1, succinic acid; M4, fumaric acid; and M24, sorbitol (*p*-value < 0.01) (Table S16). Obviously, oxalic acid had different relationships with various modules as compared with other acids. The main reason to explain this is that there is a close relationship among citric acid, fumalic acid, malate, and succinic acid in the tricarboxylic acid cycle, except for the oxalic acid [20].

### 2.5. Deciphering Key Co-Expression Modules

**Figure 7:**
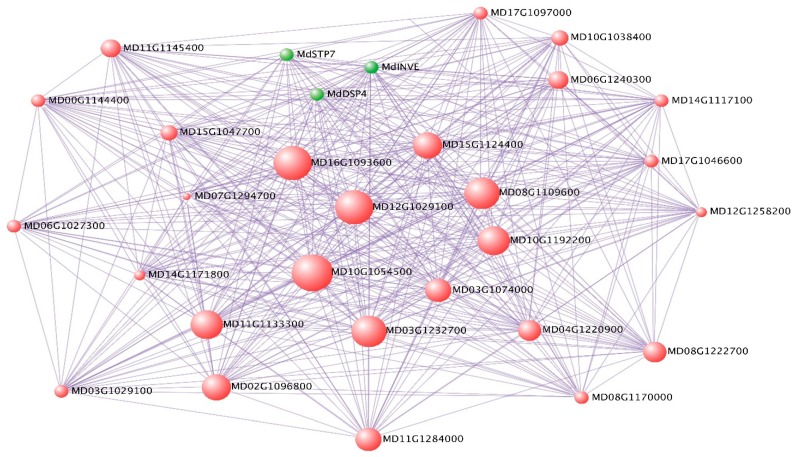
Network visualization of top 30 hub genes of co-expression module M13.

In the 331 genes of M13, 19 DEGs between Jonathan and Sweet Jonathan were detected, three (MD15G1312700, MD15G1158200, and MD00G1120100) of which were important hub genes involved in sugar accumulation during fruit development. Gene significance analysis revealed that all three genes have a highly significant relationship (*p* < 0.01) with the five sugar traits (sorbitol, glucose, fructose, sucrose, and total sugar) (Table S17 and Figure 7). *Md*DSP4 (MD15G1312700) encodes phosphoglucan phosphatase DSP, which is a type of starch granule-associated phosphoglucan phosphatase involved in the control of starch accumulation and acts as a major regulator in the initial steps of starch degradation at the granule surface. *Md*DSP4 exhibited significantly higher expression in Jonathan than in Sweet Jonathan at 90 days after bloom (90DAB) and 140 days after bloom (140DAB). *Md*STP7 (MD15G1158200), which encodes a sugar transport protein and can mediate the active uptake of hexoses by a sugar/hydrogen symport, was more highly expressed in Sweet Jonathan than in Jonathan. *Md*INVE (MD00G1120100) exhibited significantly higher expression in Sweet Jonathan than in Jonathan. These results suggest the importance of the three genes during fruit development. Besides the sugar accumulation-associated genes, genes annotated as responding to stimulation and hormones were also involved. *Md*NPR1 (MD05G1256300), which encodes regulatory protein NPR1, which is a key positive regulator of the SA-dependent signaling pathway that negatively regulates the JA-dependent signaling pathway and plays a very important role in plant stress response, had significantly higher expression in Sweet Jonathan across all fruit development stages [21,22].

A total of 12,732 genes were harbored in the largest co-expression module M26. GO enrichment analysis revealed that genes involved in glycolysis metabolism or GTP-associated signal transduction were enriched, especially the 2,198 genes identified as having a high significance (*p* < 0.01) for all five sugar traits (Figure S2). All of the above suggested the important role of glycolysis metabolism in sugar accumulation during fruit development. In addition, the especially enriched cellular response to stimulus or response to stress GO terms also showed a potential strong connection between sugar accumulation and stress (Figure S2). In the top 100 hub genes, up to 20 genes were related to plant stress or response to stress, for example, disease resistance genes, leucine-rich repeat (LRR) receptor-like protein kinase, and serine/threonine-protein kinase (Table S18). Signal transduction genes and development process-related functional genes and transcript factors were also included, for example, in the top 100 hub genes, six DEGs were included: *Md*LRP1(MD11G1186100) can function as a transcription activator that binds DNA on 5’-ACTCTAC-3’ and promotes auxin homeostasis-regulating gene expression [23,24]; *Md*bHLH70 (MD07G1160700) is a homolog of *At*bHLH70 and can play a role in the development process or stress response process [25,26]; *Md*PDCB1(MD14G1211200), and *Md*F8H is associated with cell wall development; *Md*PID could function as a serine/threonine-protein kinase involved in the regulation of auxin signaling, act as a positive regulator of cellular auxin efflux and regulates organ development by enhancing polar auxin transport [27] (Table S18).

In the top 100 hub genes of M19, except for the genes especially enriched in hydrolase activity and binding GO terms, two ATP-binding cassette (ABC) transporter C family members (MD05G1275500 and MD05G1187600) with the highest connectivity had significantly higher expression in Sweet Jonathan than in Jonathan (Figure S3 and Table S19). The top 30 hub gene *Md*SLK2 (MD02G1101700) also had higher expression in Sweet Jonathan (Table S19). SLK2 genes play an important role in response to abiotic stress and some developmental processes [28,29]. *Md*FER (MD04G1176400), with the third highest connectivity, encoding FERONIA receptor-like kinase is reported to participate in multiple process, especially in defense response against fungus and in the abscisic acid-activated signaling pathway, root development, and regulation of cell growth. Another regulator gene in the top 30 hub genes, *Md*SAB (encoding the protein SABRE), with significantly higher expression in Sweet Jonathan were annotated to negatively regulate the ethylene-activated signaling pathway and multicellular organism development [30].

Interestingly, compared with other co-expression modules, DEGs in the co-expression module M8 occupied a much larger proportion; nearly 50% (549) of all (1,153) genes in the module exhibited significant differential expression in at least one stage (Figure 5 and Table S21). At the same time, M8 had a significantly high correlation with citric acid, malic acid, fumaric acid, and total acid (*p*-value < 0.05) (Table S20 and Figure 6). All of the above suggested that the co-expression module M8 plays an important role in malate accumulation during the apple fruit development, or the acid content in the apple fruit may especially affect the expression of the genes in M8. Signal transduction and gene regulation-related genes were significantly enriched (Figure S4). In the top 100 hub genes, disease resistant genes or disease resistance regulatory genes were more abundant, for example, one homolog of WRKY19 (MD10G1007300) and one homolog of WRKY16 (MD04G1200400) were significantly more highly expressed in Sweet Jonathan than in Jonathan in all the developmental stages, while another homolog of WRKY19 (MD05G1125700) with a lower connectivity expressed higher in Jonathan (Table S21). *Md*LUG (MD08G1138900), the homolog of *At*LUG, had significantly higher expression in Jonathan as compared with Sweet Jonathan. The LUG gene participates in multiple biological processes including negative regulation of the transcription of disease-resistance genes [28].

### 2.6. Sugar Metabolism and Accumulation in Apple Fruit Development

We investigated and compared the expressed keys genes involved in sugar metabolism and accumulation in apple fruit development.

**Figure 8:**
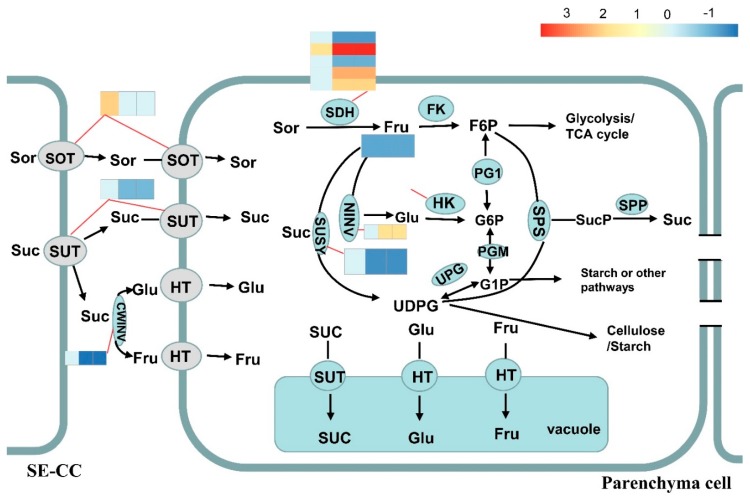
Sugar metabolism and accumulation in apple fruit.

Sorbitol is taken up into the cytosol of parenchyma cells by sorbitol transporter (SOT) [12]. We identified five genes (MD00G1152400, MD06G1148200, MD11G1003400, MD16G1055800, and MD16G1056100) expressed across the fruit development. All five genes decreased with fruit development. MD11G110340 with a high expression level (average FPKM = 46.31) across fruit development exhibited significantly higher expression in Sweet Jonathan than in Jonathan at 30 DAF, whereas no significant differential expression between the two lines could be identified in the other four genes with a very low expression level (average FPKM < 1). Whole-genome sequencing and Sanger sequencing validated that there were no destructive mutations in the CDS of the five genes between the two lines (Figure 8, Figures S5–S7, and Tables S22 and S23).

Sucrose is directly transported into parenchyma cells by sucrose transporters (SUT or SUC) located on the plasma membrane or is converted to Glc and Fru by cell invertase (CWINV) first, and, then, transported into parenchyma cells by hexose transporters [7]. Among the five expressed SUC genes, three genes with a lower expression level exhibited consistent expression, and there was no significant differential expression across all fruit developmental stages. The expression of MD15G1394000 increased drastically after 90 DAB and there was no differential expression between the two lines. Interestingly, the key expressed member (MD08G1209900) with the highest expression deceased sharply after 30 days after bloom (30 DAB) and the expression in Jonathan was significantly higher than in Sweet Jonathan (Figures S5–S7 and Table S22). With respect to the two expressed CWINV genes (MD12G1028200 and MD09G1192100), the expression of MD12G1028200 was significantly higher in Jonathan than in Sweet Jonathan after 90 DAB (Figure 8, Figure S5, and Tables S22 and S24).

**Figure 9:**
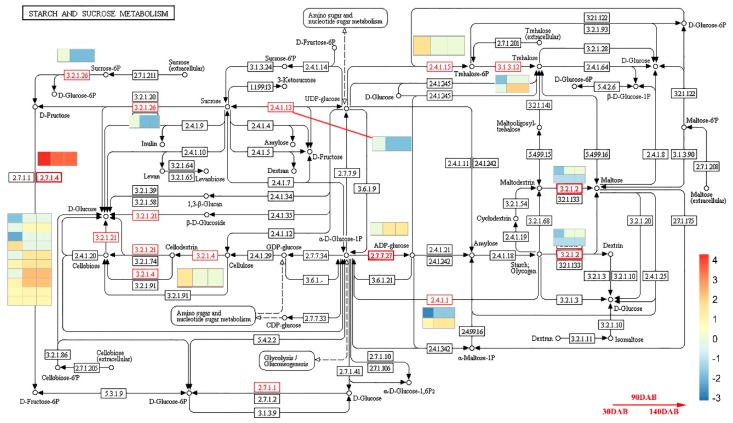
Starch and sucrose metabolism pathway. Heatmap shows the log2Foldchange (Sweet Jonathan vs. Jonathan) of the differentially expressed genes.

Sucrose is transported into sink cells (e.g., fruit, root, and shoot tips) and is converted to Fru and Glc by neutral invertase (NINV, EC 3.2.1.26) or to Fru and UDP-glucose (UDPG) by sucrose synthase (SUSY, EC 2.4.1.13) [7]. There were no destructive mutations identified in the nine expressed NINV genes, and the expression of only one (MD00G1120100) in Sweet Jonathan was significantly higher in Jonathan, at 90 DAB and 120 DAB (Figure 8 and Figure 9, Figure S5, and Table S22). For the 10 SUSY genes expressed during fruit development, the expression of one gene (MD11G1307000) with highest expression level increased with fruit development, however, that of other genes decreased dramatically after 30 DAB (Figure S5 and Table S22). The expression of two hexose transporter (HT) family genes, tonoplast monosaccharide transporter (TMT), and vacuolar glucose transporter (vGT, corresponds to massive accumulation of Glc and Flu in the vacuole. In this study, 2 vGT (MD01G1235500 and MD07G1308300) genes were expressed consistently, during fruit development. Two TMT genes (MD02G1109800 and MD02G1112900) with low expression were only expressed at 30 DAB, with no significant differential expression between the two lines, and for others (MD06G1005100 and MD10G1142100) with higher expression, the expression increased with fruit development in Jonathan but decreased at 90 DAB in Sweet Jonathan (Figure 8 and Figure 9, Figure S5, and Tables S22 and S23).

G1P is used for starch synthesis and both F6P and UDPG can be combined to resynthesize Suc via sucrose phosphate synthase and sucrose phosphate phosphatase (SPP) [7]. As for the six expressed SPS and three SPP genes, no significant difference in expression or destructive mutations was identified between the two lines (Figure 8 and Figure 9 and Table S22).

Sorbitol dehydrogenase is encoded by SDH genes and converts fructose to sorbitol. For the nine SDH genes, no destructive variations were detected in the CDS, while five genes were expressed significantly between two accessions in at least one developmental stage. One primary expression member (MD07G1054400) with significantly higher expression (average FPKM = 314.9) and two others (MD10G1062300 and MD05G1054800) had significantly higher expression in Sweet Jonathan as compared with Jonathan after 30 DAB. MD01G1195500 and MD02G1264100, with lower expression (average FPKM = 2.21, 16.21), exhibited a significant expression difference between the two accessions, at 60 days after bloom (60 DAB) and 120 DAB. All three primary expression genes (MD01G1195200, MD00G1006200, and MD07G1054400) decreased in expression during the developmental stages, while MD07G1054400 had significantly higher expression during the apple fruit development (Figure 8 and Figure 9, Figure S5, and Table S22).

Aldehyde reductase (AKR10B) converts sorbitol to glucose [7]. In this study, two genes (MD02G1264100 and MD07G1054400) with quite different expression patterns and expression levels were identified. With respect to MD02G1264100, fruits at DAB30 had high expressions levels that decreased at DAB60 and DAB120, while the MD07G1054400 expression levels increased significantly during the fruit developmental stages. MD07G1054400 (average FPKM = 314.90) exhibited significantly higher expression than MD02G1264100 (average FPKM = 16.22) across all developmental stages (*p* < 0.01). On the basis of a comparison of the gene expression levels of the two genes between Jonathan and Sweet Jonathan, both genes had higher expression levels at DAB90 and DAB120, and this is consistent with the higher content of sorbitol and glucose in Sweet Jonathan. Resequencing and Sanger sequencing suggested that there were no destructive mutations both in CDS or cis-regulatory elements between the two lines (Table S23).

Glc and Fru are phosphorylated to glucose 6-phosphate (G6P) and fructose 6-phosphate (F6P) by hexokinase (HK, EC 2.7.1.1) and fructokinase (FK, EC 2.7.1.4), respectively [7]. Among seven genes encoding hexokinase expressed across the developmental stages, MD15G1197000 exhibited the highest expression level (average FPKM = 42.59). MD03G1170700, which had the lowest expression level (average FPKM = 0.89), had significantly higher expression in fruits of Jonathan than those of Sweet Jonathan. For all seven genes, resequencing suggested that there were no destructive mutations in CDS or cis-regulatory elements between the two lines. There was no expression difference between the two lines with respect to the six expressed genes encoding fructokinase. Three distinct expression patterns were present. The expression of MD00G1004100 increased with fruit development and decreased after 90 DAB. The expression of MD01G1193000, MD04G1042400, MD09G1262800, and MD02G1197500 decreased sharply with fruit development. For MD17G1257700, there was no significant expression difference between different the fruit developmental stages (Figure 8 and Figure 9, Figure S5, and Tables S22 and S23).

PFK1 genes encode ATP-dependent 6-phosphofructokinase 1 and are involved in the synthesis of beta-D-Fructose 6-phosphate from beta-D-Fructose 1,6-bisphosphate. For all 11 expressed PFK1 genes, there were no destructive mutations in CDS between two lines, however, three genes had significantly higher expression in Sweet Jonathan than in Jonathan. Consistently, one gene (MD10G1024400) encoding fructose-1,6-bisphosphatase I, which dephosphorylates beta-D-fructose 1,6-bisphosphate and synthesizes beta-D-fructose 6-phosphate, exhibited significantly higher expression in Jonathan at 30DAB. Six FBA genes encoding fructose-bisphosphate aldolase (catalyzing beta-D-fructose 1,6-bisphosphate to D-glyceraldehyde 3-phosphate) and one other gene (MD13G1033400) exhibited significantly higher expression in Sweet Jonathan than in Jonathan, and the expression of this gene increased with fruit development (Figure 8, Figure S5, and Tables S22 and S23).

Heatmap shows the log2Foldchange (Sweet Jonathan vs. Jonathan) of the differentially expressed genes. Both sorbitol (Sor) and sucrose (Suc) are unloaded to the cell-wall space between the sieve element-companion cell complex (SE-CC) and parenchyma cells in fruit. Sor is taken up into parenchyma cells via a sorbitol transporter (SOT). Suc is directly transported into parenchyma cells by a plasma membrane-bound sucrose transporter (SUT) or is converted into fructose (Fru) and glucose (Glc) in the cell-wall space by cell-wall invertase (CWINV) and is then transported into the parenchyma cells by a hexose transporter (HT). In the cytosol, Sor is converted to Fru by sorbitol dehydrogenase (SDH), while Suc is converted to Fru and Glc by neutral invertase (NINV), or to Fru and UDP-glucose by sucrose synthase (SUSY). The resulting Glc and Fru can be phosphorylated to glucose 6-phsophate (G6P) and fructose 6-phosphate (F6P) by hexokinase (HK) and fructokinase (FK, specific for Fru). The conversions between F6P, G6P, G1P, and UDPG are catalyzed by phosphoglucoisomerase (PGI), phosphoglucomutase (PGM), and UDPG-pyrophosphorylase (UGP) in readily reversible reactions. The F6P produced in sugar metabolism enters the glycolysis/TCA cycle to generate energy and intermediates for other processes. G1P is used for starch synthesis. UDPG can be used for cellulose synthesis or combined with F6P for resynthesis of Suc via sucrose phosphate synthase (SPS) and sucrose-phosphatase (SPP). Most of the Fru, Glc, and Suc that have not been metabolized are transported by special tonoplast transporters into the vacuole for storage. Inside the vacuole, Suc converted into Glc and Fru by vacuolar acid invertase (vAINV).

### 2.7. The Stop Gain Mutation in MdMa1 may Partly Explain the Low Acid Content in Sweet Jonathan

We identified nine expressed malate transporter genes and two genes (MD06G1096000 and MD14G1116200) that had significantly higher expression in Sweet Jonathan than in Jonathan at 90 DAB and 140 DAB. A stop-gain (A- > G) mutation was identified in the CDS of one gene (MD13G1044400). Ma1 (MD16G1045200) was reported to be the candidate genes controlling the malate content [31]. In this study, Ma1 was expressed at a much higher level than the other eight genes (Table S25). Whole genome resequencing and Sanger sequencing identified and validated the existing mutation leading to a premature stop codon that truncates the carboxyl terminus of Ma1-1455A by 84 amino acids as compared with Ma1-1455G in Sweet Jonathan. The existence of a stop-gain mutation in the coding sequencing of Ma1 may be one of the important causes of the lower malate content in Sweet Jonathan.

### 2.8. Dramatic Upregulation of ABC Transporter Genes in Sweet Jonathan is Potentially Regulated by MdBPC6

**Figure 10:**
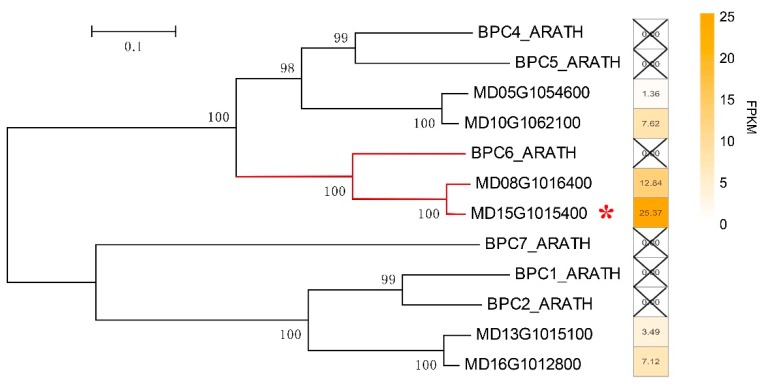
Phylogenetic analysis of the BPC gene family in the apple genome. The heatmap indicates the average fragments per kilobase of transcript per million mapped (FPKM) values of all samples. * the gene with the highest expression

ATP-binding cassette (ABC) transporter proteins are known to play many important roles in the physiology and development of plants and have been linked to the enhancement of crop yield and stress tolerance [12]. Among the 281 expressed ABC transporter genes, 191 genes had a very low expression level (FPKM < 1). Among the other genes with higher expression (FPKM > 1), 17 exhibited differential expression in at least two stages across fruit development. Interestingly, which 14 genes were expressed at a significantly higher in Sweet Jonathan than in Jonathan, especially at 90 DAB and 140 DAB. Furthermore, we conducted motif analysis based on the upstream (2000 bp) sequences of the 17 DEGs and obtained one consensus motif, which was significantly (*p*-value < 0.01) similar to the GA-rich binding site element of BPC6 (MA1402.1) (Figures S9a and S10 and Table S26). In total, six expressed homologs of BPC genes were identified in the apple genome, and only the primary expression member MD15G1015400 with the highest expression was expressed at a significantly higher level in Sweet Jonathan than in Jonathan across the fruit development (Figure 10 and Figure S9b).

**Figure 11:**
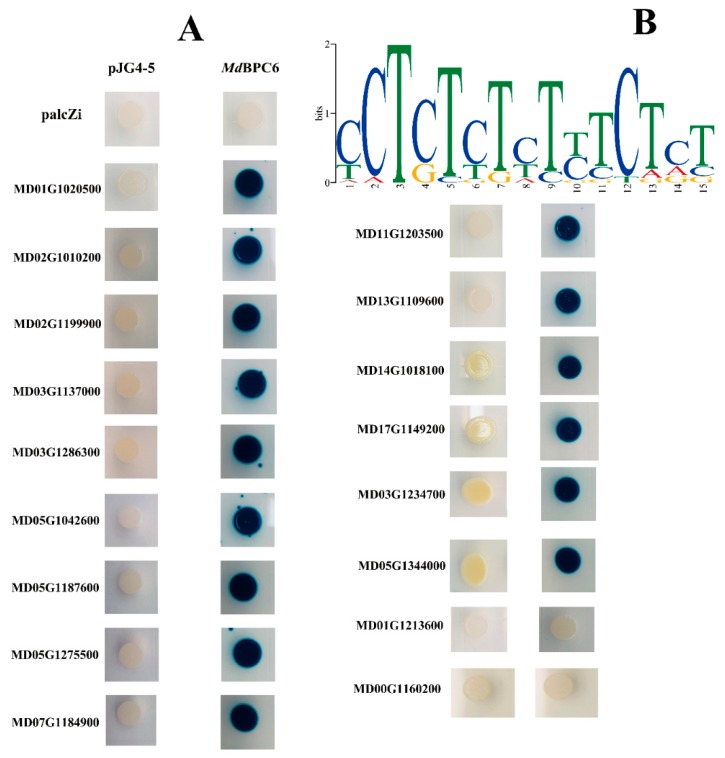
Binding ability and transcriptional activation assays for *Md*BPC6 with ABC transporter genes. (**A**) Interaction of the *Md*BPC6 protein with the promoters of ABC transporter genes based on yeast one-hybrid assays. The panel shows yeast cells containing distinct effector and reporter constructs grown on an SD/-Trp/-Ura medium plate. Interaction of *Md*BPC6, fused to the GAL4 activation domain (pJG4-5-*Md*BPC6), with LacZ driven by the ABC transporter gene promoters (pLacZi-promoter of ABC transporter genes) is shown in the left top panel. Yeast transformed with pJG4-5-*Md*BPC6/pLacZi, pJG4-5/pLacZi-promoter of ABC transporter genes, and pJG4-5/pLacZi were used as controls. (**B**) Cis-regulatory elements in upstream gene sequences identified from 17 differentially expressed ABC transporter genes using MEME [32].

The GA-rich element of *Md*BPC6 binds to the promoter regions of 17 differentially expressed ABC transporters genes. Yeast one-hybrid assay validated that 15 of the 17 putative ABC transporters genes interacted with the *Md*BPC6 protein (Figure 11). The unvalidated two genes suggested that the MdBPC6 would not bind to the promoters of the two genes and the consensus motif should be false discovery.

### 2.9. PlantHhormone Signal Transduction

**Figure 12:**
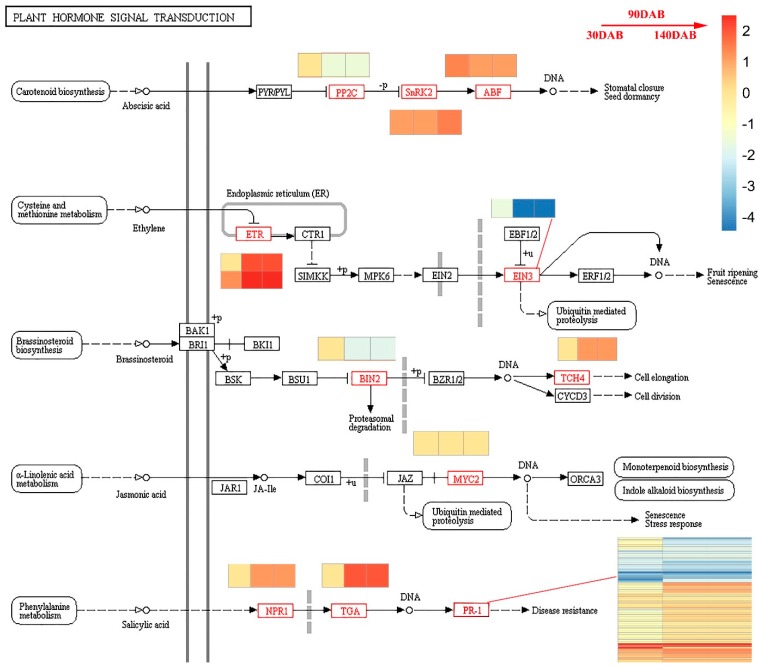
Plant hormone signal transduction during apple fruit development. Heatmap shows the log2Foldchange (Sweet Jonathan vs. Jonathan) of the differentially expressed genes.

Plant hormone signal transduction is closely related to fruit development and plant stress. Here, differentially expressed genes were enriched in signal transduction GO terms, especially hormone signal transduction (*p* < 0.01) (Tables S11–S13). Fourteen DEGs and two DIGs were investigated in the plant hormone signal transduction pathway (mdm04075) (Table S27 and Figure 12). A series of successional DEGs in ABA and SA pathways were identified. One gene (MD11G1093100) in the ABA pathway encoding protein phosphatase 2C (PP2C; K14497), which negatively regulated serine/threonine-protein kinase SRK2, had significantly higher expression in Jonathan than in Sweet Jonathan, whereas MD04G1054400 encoding serine/threonine-protein kinase SRK2 and another gene (MD05G1027000) encoding an ABA responsive element binding factor had significantly higher expression in Sweet Jonathan. In the SA pathway, one gene (MD05G1256300) encoding regulatory protein NPR1 (K14508), which positively regulates transcription factor TGA (K14431), had significantly higher expression in Sweet Jonathan, and consistently, MD13G1044600, which encodes TGA, was upregulated in Sweet Jonathan (Table S27).

### 2.10. Disease Resistance Protein Genes

Sugar in plants may play a role in regulating plant disease resistance. During fruit development, defense resistance-related genes, especially disease resistance genes, were obviously observed significantly enriched (*p* < 0.01) (Tables S11–S13). Of the identified 765 disease resistance genes, 639 genes were expressed at a very low level (average FPKM < 1). Thirty disease resistance genes were differentially expressed in at least two stages between Jonathan and Sweet Jonathan of which 21 genes were expressed at a higher expression level in Sweet Jonathan (Table S28 and Figure S10).

## 3. Discussion

### 3.1. Huge Numbers of Mutations Identified by Whole Genome Resequencing Suggested Variable Levels of Genetic Diversity among Apple Bud Mutant Cultivars

A significant number of domesticated plants including apple, banana, potato, grape, and coffee are vegetatively propagated to maintain agronomically valuable genotypes, however, after many propagation cycles, clones accumulate phenotypic differences in agronomic traits and clonal diversity appears. In the case of long-lived plants, it has been argued that because of the large numbers of somatic cell divisions separating zygote from gamete formation, a significant number of somatic mutations, in theory, can accumulate within the cell lineages that eventually differentiate into gametogenic tissue. Even for the same cultivar, millions of variations could also be detected, possibly if sampled from different geographic positions, for example, 3,498,958 SNPs and 407,982 InDels were reported to be identified between “Golden Delicious” and apple reference genome (also Golden Delicious) [33]. In addition, similar work has also been conducted by the Research Institute of Pomology, Shandong Academy of Agricultural Sciences. For two cultivars, “Fuji” and its bud mutant cultivar “YanFu1”, high coverage (~50x) resequencing and variations analysis revealed that there were 1,893,212 SNVs between two cultivars (personal communication, April 15, 2018). Carrier et al. performed genome sequencing and identified huge numbers of SNVs and SVs among somatic mutation cultivars in grapevine [34]. Lee et al. detected 2,820,759 of SNVs in Fuji apple somatic variants using next-generation sequencing [35]. In this study, we first conducted an average of 50× whole genome resequencing of Jonathan and Sweet Jonathan, and a total of 4,198,955 SNPs and 319,494 InDels were identified (Table S3). Among them, 5,487 destructive mutations, which will cause destructive effects on genes, were identified in the CDS of 3,872 genes (Tables S4 and S5). A total of 32,434 SVs were detected. Large segment deletions and insertions located in either the exonic region of genes (1,035) or the upstream regions of genes (3,701) could lead to a serious impact on gene function (Table S7). Gene functional analysis revealed that the influenced genes were related to upregulation of most of the metabolic pathways. The above-mentioned results suggest there may be a higher number of variations among bud mutant cultivars and many genes may be involved in the phenotypic differences.

### 3.2. Identifying Hub Genes Associated with Sugar and Acid Accumulation by Deciphering Co-Expression Modules

Deciphering of co-expression modules provided a viable means to identify candidate key genes for sugar and acid accumulation. Genes playing a more important role and with large phenotypic variation have a higher possibility to be hub genes in intra co-expression networks. Bai et al. uncovered co-expression gene network modules regulating fruit acidity in diverse apples and identified the *Ma* gene placing in the top spot of the intra co-expression module, as well as key regulatory genes associated with apple acid accumulation [10]. El-Sharkawy et al. performed transcriptome analysis of an apple (*Malus domestica*) yellow fruit somatic mutation and identified a gene network module harboring MYB10 that was strongly associated with anthocyanin and epigenetic regulation [3]. Yang et al. analyzed a co-expression network and identified SPL TFs as the candidate regulatory genes associated with anthocyanin accumulation in Crabapple leaf color transformation [36]. Here, by using 24,047 genes with more variable expression values across fruit development, we constructed 28 co-expression modules and 14 trait-associated co-expression modules were identified. By deciphering co-expression modules, many hub genes potentially associated with sugar and acid accumulation were identified. In M13, three genes with the highest connectivity among the intra network, *Md*DSP4 (MD15G1312700), *Md*STP7 (MD15G1158200), and *Md*INVE (MD00G1120100), which were annotated to be associated with starch accumulation, sugar transporting, and fructose accumulation, respectively, were speculated as key genes controlling sugar accumulation during fruit development (Table S17). In M26 and M19, regulatory factors and functional genes were also identified, for example, *Md*bHLH70, *Md*PDCB1, and *Md*F8H2 could play a role in regulating developmental processes and cell-wall development, respectively, which may directly or indirectly influence the sugar content or flavor [25,26] (Table S18). Substance-specific modules indicate the existence of specific regulatory genes or key genes in the associated module. In succinic acid specific module (M1), we identified one transcript factor *Md*BZP53 (MD11G1314100), the homolog of *At*BZP53, which were previously reported to be associated with amino acid metabolism and stress [37]. In the 159 hub genes of the fumaric acid specific module (M4), we also found the following three TFs: *Md*RF2B (MD01G1167000), *Md*bHLH140 (MD04G1160300), and MdbHLH62 (MD10G1156100). Sorbitol should be one of the most important substance in sugar synthesis and metabolism in the *Rosidea* family and was also reported to be associated with plant hormone synthesis and transduction in apple [38,39]. In the Sorbitol specific module (M24), among the 59 hub genes associated with sorbitol accumulation, we found two genes, MD12G1227200 (Gibberellin receptor) and MD12G1118200 (Auxin response factor), involved in plant hormone signal transduction. All of the identified genes could provide a reference for studying the gene regulatory network associated with sugar and acid accumulation.

### 3.3. Sugar and Acid in Apple Fruit may Influence Stress during Fruit Development

The sugar and acid in apple fruit may actively influence stress during fruit development. Sugars produced by photosynthesis not only fuel plant growth and development but may also act as signals to regulate plant growth and development, as well as plant stress. In this study, although there was no significant difference between the total sugar of Jonathan and Sweet Jonathan during fruit development, the important sugar components showed significant differences, for example, the sorbitol content in Sweet Jonathan was significantly higher than in Jonathan. Suppressing sorbitol synthesis could substantially alter the global expression profile of stress response genes in apple and influence disease resistance [12,17]. Here, in the sugar-associated modules, hub genes involved in sugar metabolism were identified, and basic genes associated with plant stress were enriched or were more abundant in the same module (Tables S17–S19 and S21), for example, M13 was associated with all five types of sugar components and, consistently, three genes (*Md*DSP4, *Md*STP7, and *Md*INVE) associated with sugar accumulation were identified as huge genes; in the same module, *Md*NPR1, which is a key regulatory gene associated with the SA-pathway and disease resistance, exhibited significantly higher expression in Sweet Jonathan than in Jonathan, consistent with the fact that disease-resistance genes in Sweet Jonathan were more highly expressed during fruit development (Table S17). In M26, besides the higher abundance of the disease resistant genes, *Md*FER (MD04G1176400) with the third highest connectivity, encoding the FERONIA receptor-like kinase, was reported to participate in defense response to fungus (Table S18).

Malate accumulation in fruit is strongly influenced by environmental factors, such as temperature, mineral availability, drought, and salt stresses [40,41,42]. In turn, malate accumulation could respond to and moderate stress in fruits such as tomato, grapevine, citrus, and melon [11,42]. In this study, malate in Sweet Jonathan was significantly lower than that in Jonathan across all fruit developmental stages. The key co-expression module, M8, harbored the highest proportion of DEGs and exhibited a significantly high correlation with citric acid, malic acid, fumaric acid, and total acid (Tables S17 and S20). Disease resistance regulatory genes were identified in the hub genes, including *Md*WRKY19, *Md*WRKY16, and *Md*LUG. The expression of regulatory genes was consistent with the higher expression of the disease related genes in Sweet Jonathan (Table S21).

Collectively, deciphering genes in co-expression modules suggest that sugar and acid (especially sorbitol and malate) in apple fruit potentially influence stress associated with genes directly or indirectly.

### 3.4. TranscriptFfactor MdBCP6 Could Regulate ABC Transporter Genes and Potentially Participate in Fruit Development or Stress Response

Apple fruit development is a complex process regulated by numerous TFs, for example, *Md*MYB10 regulates red coloration in apple fruit, and *Md*MYB1 regulates malate accumulation during fruit development. BPC TFs specifically bind to GA-rich elements (GAGA-repeats) present in regulatory sequences of genes involved in developmental processes [43]. Plant ABC transporters play important roles in pathogen responses and development [44]. In this study, by comparing the gene expression profiles of Jonathan and Sweet Jonathan during fruit development, 17 differentially expressed ABC transporter genes were identified, of which 15 showed a more consistent profile and exhibited higher expression levels in Sweet Jonathan (Figure S8). Motif-analysis and yeast one-hybrid assay identified and confirmed that *Md*BPC6 could bind to the GAGA-motif in the promoter sequences of differentially expressed ABC transporter genes and regulate the expression level (Figure 11). All of the above indicates that *Md*BPC6 could participate in apple fruit development or stress response processes by regulating ABC transporter genes.

## 4. Materials and Methods

### 4.1. Plant Materials and Sample Collection

Two cultivars “Jonathan” and its bud mutant cultivar “Sweet Jonathan” used in this study were grown at the Institute of Pomology of the Chinese Academy of Agricultural Sciences, Xingcheng, Liaoning, China. For each cultivar, three scions were grafted onto three rootstocks of the same variety (*Malus baccata* Borkh). The three seedings from each cultivar were planted in the field in 2005. To investigate the dynamic of sugar and acid accumulation during fruit development, we collected five apple fruits from the three trees of each cultivar at 30, 90, and 140 days after bloom (DAB), respectively. On the basis of the previous reports about the sugar and acid accumulation during apple fruit development, we selected three time points (30, 90, and 140 DAB) to represent three important fruit development stages, young fruit stage, fruit expansion stage, and fruit maturity stage, respectively [45,46]. For each apple, the skin-free cortex was excised, and cut into small sections, immediately frozen in liquid nitrogen, and then stored at −80 °C.

### 4.2. Measurement of Sugars and Acid

In order to obtain accurate phenotypes, we measured the sugar and acid contents of 10 fruits from the two cultivars. Each sample was ground into a fine powder in liquid nitrogen using a SPEX 6870 Freezer Mill (SPEX, Metuchen, USA). The power (10 g) was extracted with ultra-pure water. After centrifugation at 15,984× *g* for 10 min, the supernatants were purified using an OnGuard II Ag cartridge column (Dionex Corporation, Sunnyvale, CA, USA) and filtered through a 0.22 μm Water membrane Sep-Pak filter. The filtered supernatants were used to measure sorbitol, glucose, fructose, sucrose, oxalate acid, citric acid, malic acid, succinic acid, and fumaric acid using the DIONEX ICS-5000 HPLC system (Dionex Corporation, Sunnyvale, CA, USA).

We conducted the statistical analysis using Graphpad Prism (v8.2.1) software and particularly analysis of variance (ANOVA) was used to test the difference between groups.

### 4.3. Resequencing of Jonathan and Sweet Jonathan

Genomic DNA was extracted from the leaves of Jonathan and Sweet Jonathan using a Genomic DNA Isolation Kit (TianGen, Beijing, China). The illumina sequencing libraries were constructed using NEBNext DNA Library Prep Master Mix (NEB). Paired-end sequencing was performed using the Illumina HiSeq X ten sequencer (Illumina, San Diego, CA, USA). The resequencing data have been deposited in the NCBI Sequence Read Archive (SRA) under accession number PRJNA516602. Burrows-Wheeler Aligner (BWA) (version 0.7.17) was used to map filtered reads to the apple double haploid (DH) genome [46,47,48]. The BAM files were processed (including sorting, removal of duplicates, and indexing) using SAMtools software (version 1.9) [49]. SNP and InDel calling were conducted using SAMtools and BCFtools software [49,50]. Specifically, the SAMtools mpileup function was used to process BAM files and to generate BCF files. In order to reduce the influence of multiple alignment, only unique alignments (mapQ > 20) were retained in the mpileup process. SNPs and InDel were identified using the BCFtools call function and an alternative model for multiallelic calling (options, multiallelic-caller) was applied. Finally, SNPs and InDels with a lowest phred-scaled quality of 30 and a minimum read depth of 10 were retained. Structure variation calling was conducted using Delly (version 0.8.1) software [51]. Delly is an integrated SV prediction method that uses paired-ends, split-reads, and read-depth to sensitively and accurately delineate genomic rearrangements. The BAM files of the two cultivars were fed into the Delly call function with default parameters to call and genotype five types of SVs (deletion, duplication, inversion, translocation, and insertion). Cis-acting regulatory elements upstream of the genes were analyzed using PlantCARE web tools [52]. All the variations were annotated with ANNOVAR (version 20080416) based on the functional domain, cis-acting regulatory elements, and gene model information [53].

### 4.4. RNA-Seq Library Construction, Sequencing, and Data Processing

We selected three apple fruits of each cultivar (one fruit from each tree) from the collected fruits and conducted an RNA-sequencing experiment. Total RNA was extracted using the modified CTAB method [54]. For each sample, 5 μg total RNA was used to isolate mRNA for the preparation of an RNA-seq library using the NEBNext Poly(A) mRNA Magnetic Isolation Module and NEBNext Ultra Directional RNA Library Prep Kit for Illumina (New England Biolabs, Massachusetts, USA) following the manufacturers’ protocols. The cDNA library was sequenced (PE150) from both the 5’ and 3’ ends on an Illumina HiSeq X Ten platform (Illumina) according to the manufacturer’s instructions.

The RNA-sequencing data have been deposited in the NCBI Sequence Read Archive (SRA) and under accession number PRJNA516602. All raw reads were processed by Fastqc to check the read quality and adapters contamination. The raw reads were processed by NGSQC to remove low-quality reads and adapters [55]. The resulting cleaned reads were then aligned to the Malus genome using HISAT2 [56]. StingTie was used to assemble transcript and for quantification [57]. Raw counts of genes in each sample were extracted. Differentially expressed genes were detected using DESeq2 software with default parameters and genes with FDR less than 0.05 were identified as differentially expressed genes [58]. The R package VennDiagram was used to generate Venn diagrams [59].

To identify the replicates with similar expression patterns, we performed hierarchical cluster analysis (HCA) among all samples using script PtR from the Trinity package [60]. Briefly, PtR reads in the matrix of counts, performs a counts-per-million (CPM) data transformation followed by a log2 transform and generates a correlation matrix for all sample replicates. Blast2GO [61] software was used to identify GO enrichment terms. We set the threshold value of the false discover rate (FDR) to < 0.05. A directed acyclic graph and treemap of the enriched GO terms were generated by blast2GO [61] and reviGO [62]. We preformed KEGG metabolism analysis using KOBAS 2.0 [63] software.

### 4.5. Identification of Co-Expression Modules

The R package WGCNA was used to identify modules of highly correlated genes based on the FPKM data [18,19]. The R package DCGL was used to filter the genes based on gene expression and variation [64]. Specifically, we used two functions in the DCGL package: “expressionBasedfilter” to filter out genes with a Between-Experiment Mean Expression Signal (BEMES) lower than the median of BEMES’s of all genes and “varianceBasedfilter” to filter those genes not significantly more variable than the median gene (*p*-value threshold = 0.05). With the help of the function pickSoftThreshold in WGCNA package, the soft thresholding power was chosen as 8. The power was interpreted as a soft threshold of the correlation matrix. The resulting adjacency matrix was then converted to a topological overlap (TO) matrix by the TOM similarity algorithm. Genes were hierarchically clustered based on TO similarity. We used the dynamic hybrid tree cut algorithm 30 to cut the hierarchal clustering tree, and defined modules as branches from the tree cutting. We summarized the expression profile of each module by representing it as the first principal component (referred to as module eigengene). Modules whose eigengenes were highly correlated (correlation greater than 0.75) were merged. The genes in each module were then processed following the method in the GO enrichment analysis and pathway enrichment analysis.

### 4.6. Visualization of Hub Genes

Genes with the highest degree of connectivity within a module are referred to as intramodular hub genes [18]. The top 100 hub genes from each module from two conditions, ranked by KME, were selected and the hub genes in each module were compared. The top 100 hub genes of the focused module were visualized by VisANT in the network.

### 4.7. Gene Expression Analysis

To examine the RNA-seq data, randomly selected genes were validated using RT-qPCR, and the gene-specific primers were designed with Primer Premier software (version 5.0) (Premier Biosoft Interpairs, Palo Alto, CA) (Table S1). One microgram of total RNA was used in the reverse transcription in a total volume of 20 μL in the presence of 6-mer random primer and oligo primer according to the protocol of the TaKaRa kit (TaKaRa Biotechnology (Dalian) Co., Ltd., Dalian, China). The PCR reactions were run in three replicates in a Bio-Rad Sequence Detection System (Bio-Rad Life Science Research, Hercules, CA, USA). Actin was chosen as an internal control gene for normalization. Quantifying the relative expression of the genes at the three sampling time points was performed using the delta-delta Ct method [65]. All data were expressed as the mean ± SD after normalization.

### 4.8. Validation of SNPs and InDels

Primers were designed using Primer-blast software (NCBI, Maryland, USA) based on the 200-bp sequences flanking the SNPs and InDels to be validated (Table S2). Then, monoclonal PCR products comprising SNP loci of interest were amplified in both parents and sequenced using Sanger sequencing (BGI, Beijing, China) to verify the SNPs obtained from the resequencing data between the parents.

### 4.9. Identification of Cis-Motifs

The MEME program was used to identify motifs in the promoter regions of genes from each co-expression module [32]. We defined the promoter regions as 1 kb upstream and 500 bp downstream of the transcription start sites and obtained the genomic sequences using a customized Perl script. For each module, 10 motifs (motifs 1–10) were reported by MEME, and those with an E-value greater than 10 to six were manually excluded. Using the TOMTOM motif comparison tool, the resulting motifs were aligned with motifs in the JASPAR CORE Plantae database [66,67] to identify significantly similar known cis-motifs (*p*-value < 0.01).

### 4.10. Yeast One-Hybrid Assay

Yeast one-hybrid analysis was used to assay transcriptional activation by the *Md*BPC6. The open reading frame of *Md*BPC6 were cloned into the EcoRI and XhoI sites of the pJG4-5 vector (Clontech) under the control of the galactokinase 1 (GAL1) promoter, giving the effector constructs. The selected ABC transporter gene promoter sequences were inserted upstream of the reporter LacZ gene in the pLacZi vector. The effector and reporter or control constructs were transformed into competent yeast cells (Saccharomyces cerevisiae) strain EGY48, resulting in the following yeast strains: pJG4-5-*Md*BPC6/pLacZi-promoter of 17 differentially expressed ATP-binding cassette (ABC) transporter genes, pJG4-5/pLacZi-promoter of ABC transporter genes, pJG4-5-*Md*BPC6/pLacZi, and pJG4-5/pLacZi. The cells were selected on synthetic dropout media lacking tryptophan and uracil, and positive colonies were spotted onto glucose plates (2%) containing X-gal at 28 °C for 2 d to confirm blue color development [36].

## 5. Conclusions

Using two bud sport materials with different sugar and acid flavor, i.e., Jonathan and Sweet Jonathan, we profiled the whole genome variations and transcriptional regulatory network during the fruit developmental stages by whole genome sequencing and RNA-sequencing. We identified the hub genes associated with sugar and acid metabolism by deciphering co-expression modules associated with sugar or acid accumulation during fruit development.

We first deciphered the intra relationship between stress-associated genes and sugar and acid accumulation-associated genes within the consensus co-expression module. *Md*BPC6 genes, first identified as regulatory factors of ABC transporter genes, regulate apple fruit development and stress.

## Supplementary Materials

Supplementary Materials can be found at https://www.mdpi.com/1422-0067/20/23/5988/s1.
