# Supplementary material for: Parallel Bud Mutation Sequencing Reveals that Fruit Sugar and Acid Metabolism Potentially Influence Stress in Malus
Source: Int J Mol Sci. 2019 Nov 28;20(23):5988. doi: 10.3390/ijms20235988 (PMC6928686; doi:10.3390/ijms20235988)
Supplement: Supplementary file 1 [file ijms-20-05988-s001.zip › Supplementary Figures.pdf]

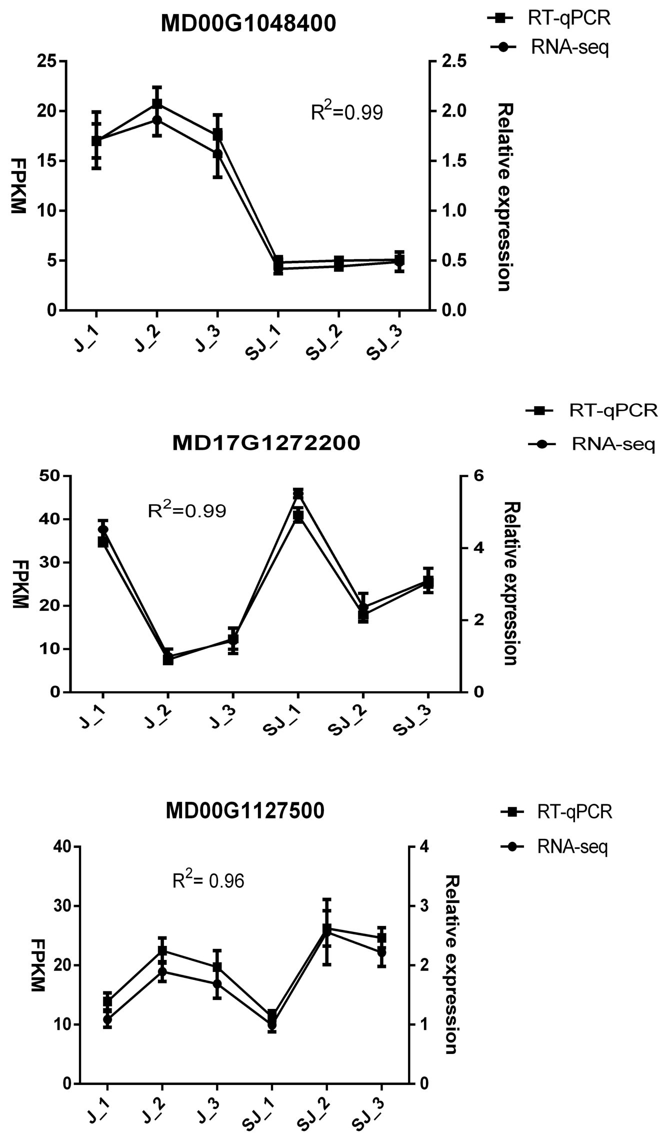

Figure S1 Correlation analysis between RNA-sequencing and RT-qPCR of three representative genes

J_1 (or SJ_1): samples from 'Jonathan' (or 'Sweet Jonathan') and sampled at 30 days after bloom (30 DAB); J_2 (SJ_2) : samples from 'Jonathan' (or 'Sweet Jonathan') and sampled at 90 days after bloom (90 DAB); J_3 (SJ_3): samples from 'Jonathan' (or 'Sweet Jonathan) and sampled at 140 days after bloom (140 DAB);

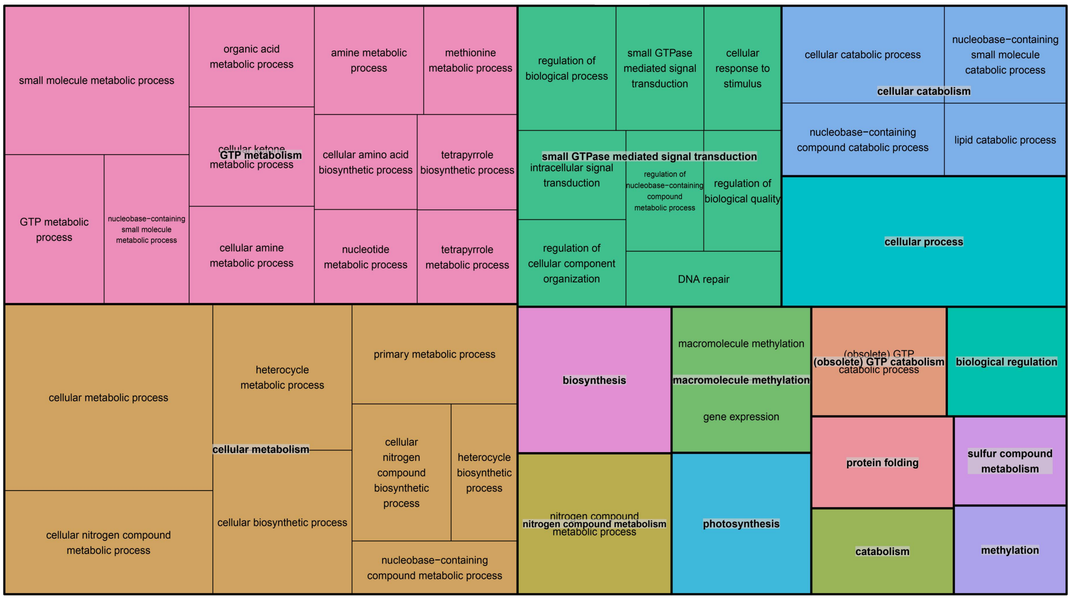

Figure S2 Treemap of the enriched gene ontology (GO) terms (P value <0.01) in co-expression module M26

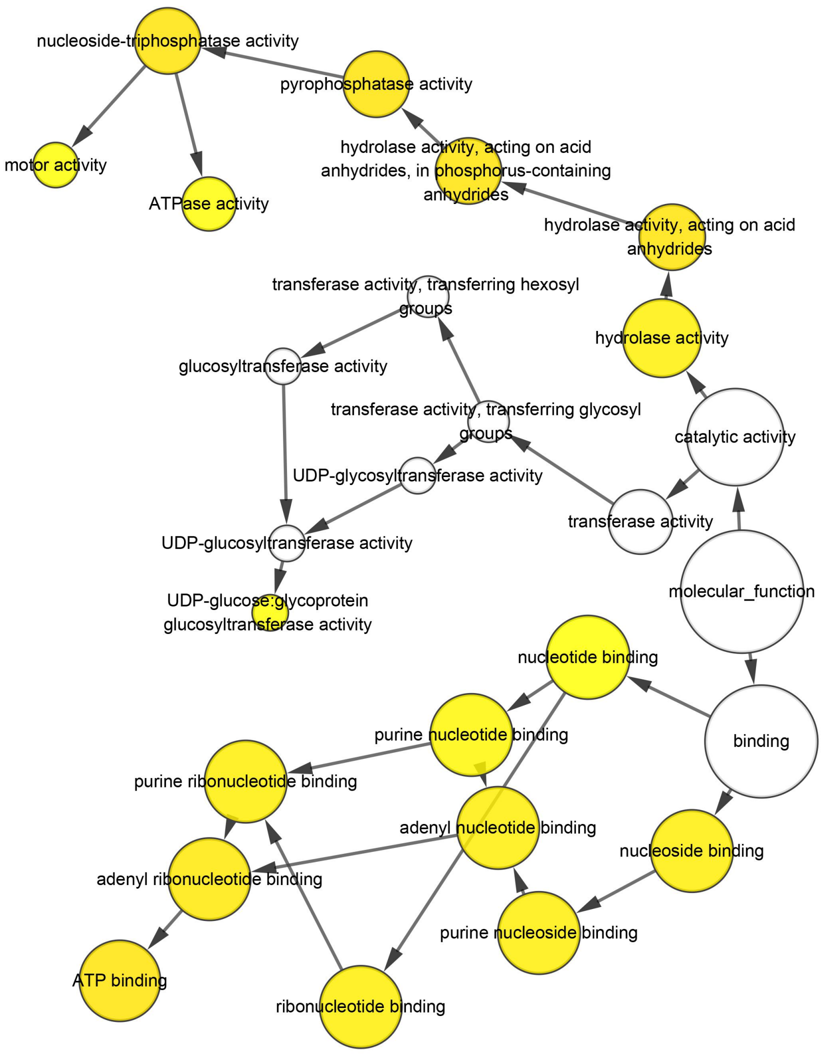

Figure S3 Enriched GO terms (P <0.05) in the top 100 hub genes of M19

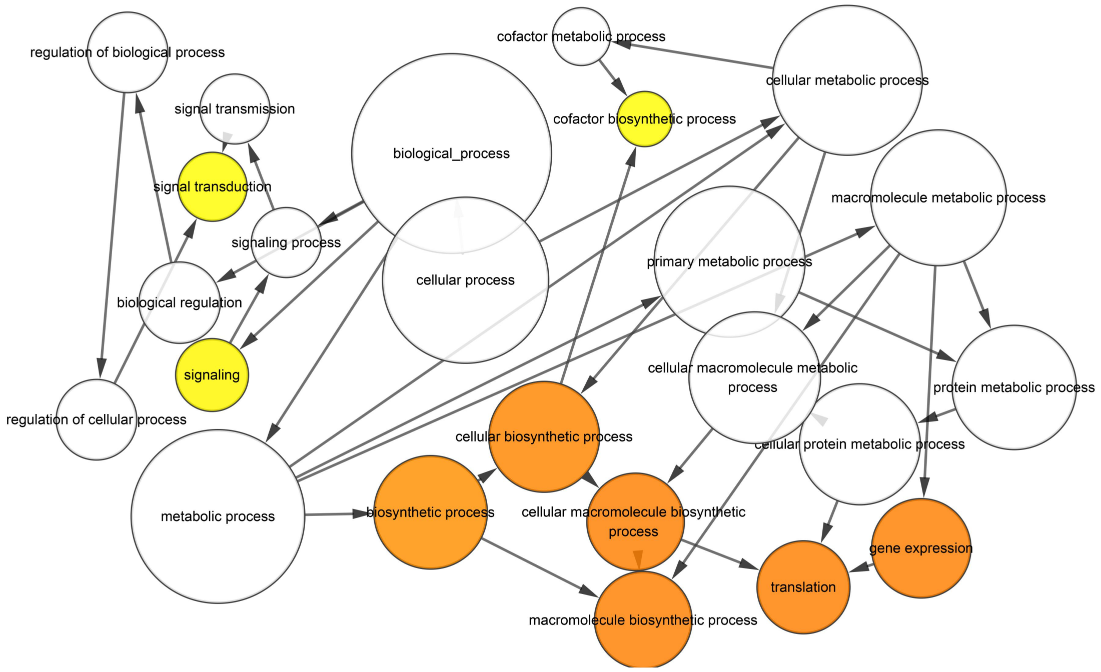

Figure S4 Significantly enriched GO terms (P value <0.05) in the co-expression M8

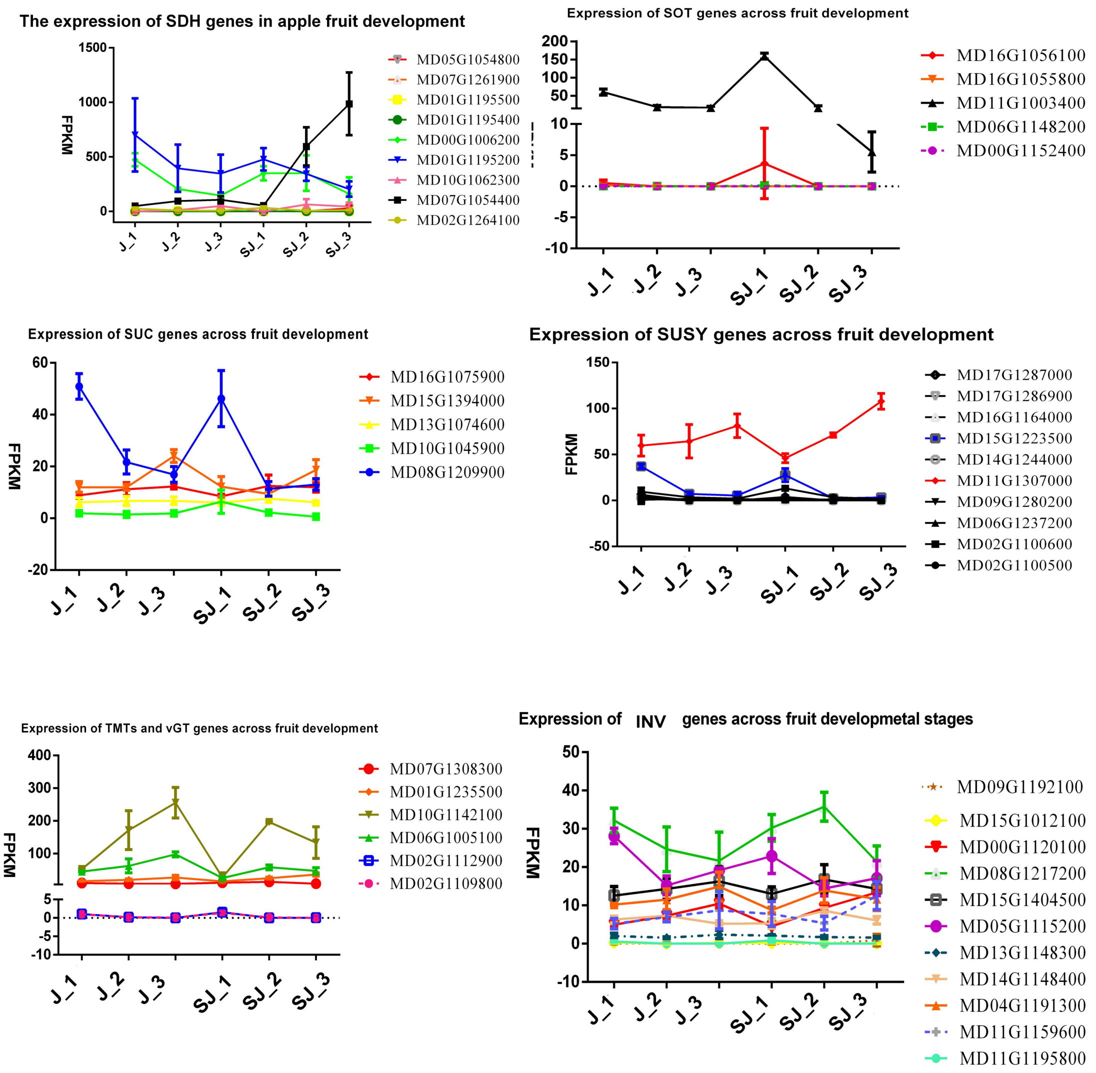

Figure S5 Expression profile of important genes in the sugar accumulation during apple fruit development process

J_1 (or SJ_1): samples from 'Jonathan' (or 'Sweet Jonathan') and sampled at 30 days after bloom (30 DAB); J_2 (SJ_2) : samples from 'Jonathan' (or 'Sweet Jonathan') and sampled at 90 days after bloom (90 DAB); J_3 (SJ_3): samples from 'Jonathan' (or 'Sweet Jonathan) and sampled at 140 days after bloom (140 DAB);

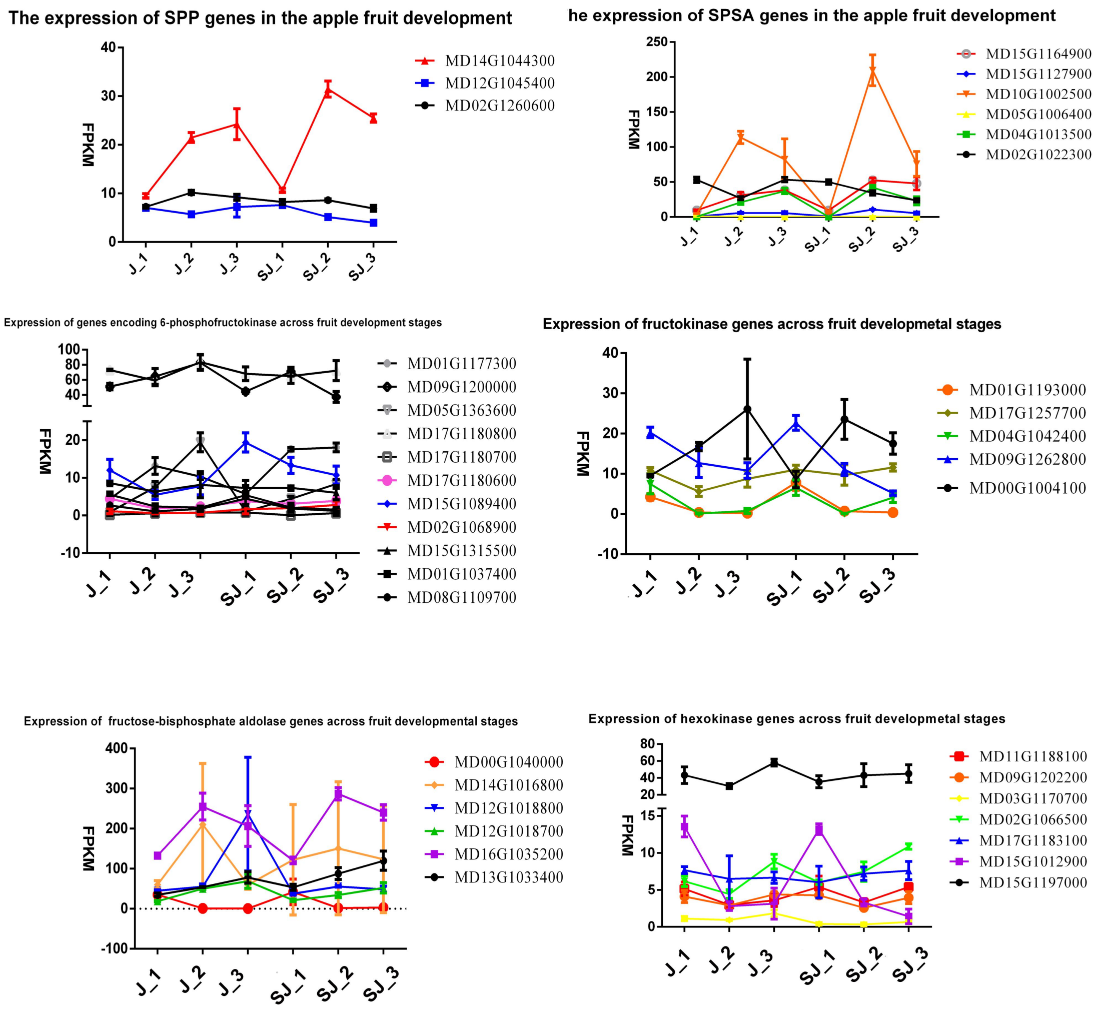

Figure S6 Expression profile of important genes in the sugar accumulation during apple fruit development process

J_1 (or SJ_1): samples from 'Jonathan' (or 'Sweet Jonathan') and sampled at 30 days after bloom (30 DAB); J_2 (SJ_2) : samples from 'Jonathan' (or 'Sweet Jonathan') and sampled at 90 days after bloom (90 DAB); J_3 (SJ_3): samples from 'Jonathan' (or 'Sweet Jonathan) and sampled at 140 days after bloom (140 DAB);

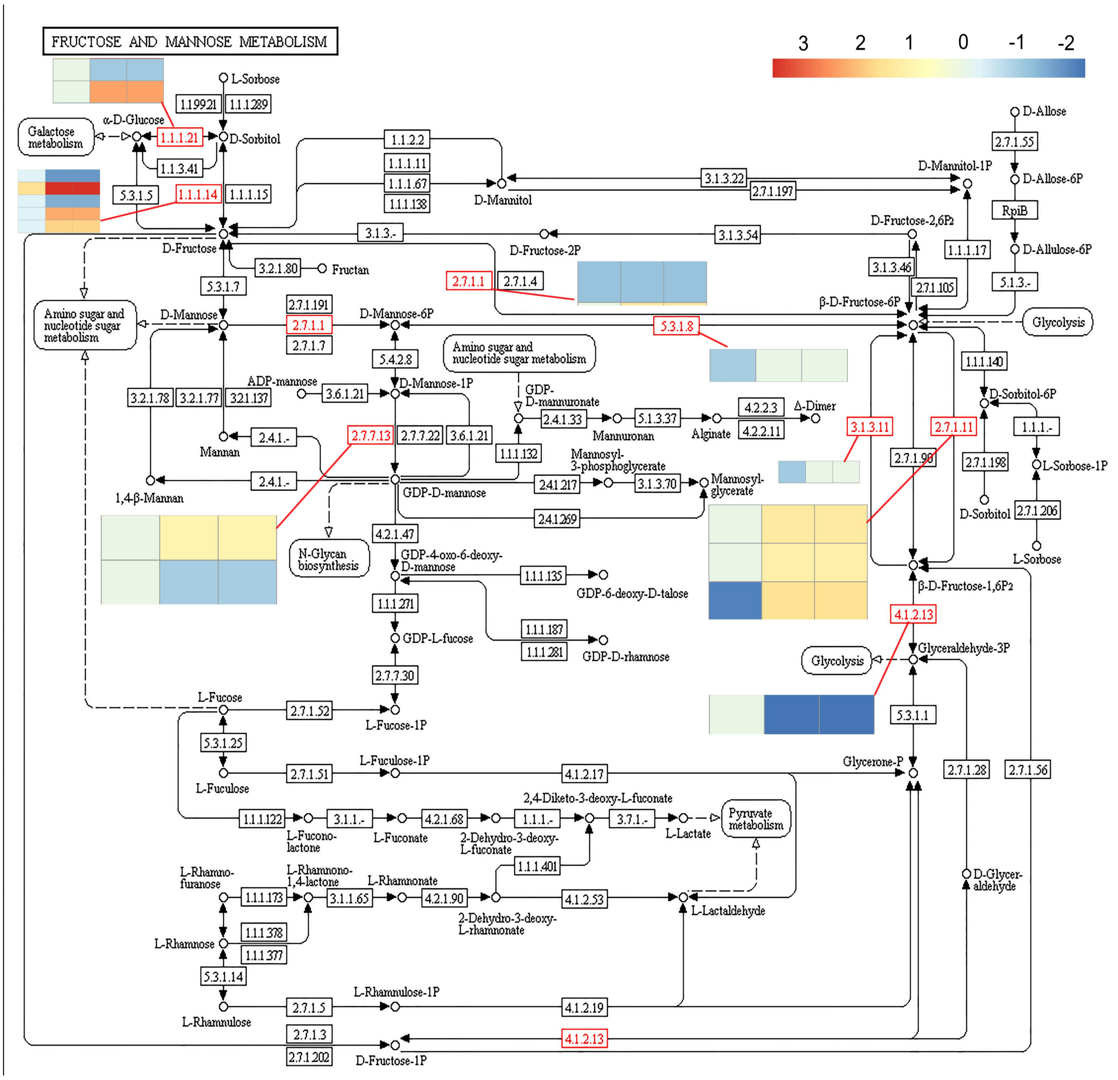

Figure S7 Fructose and mannose metabolism pathway.

Heatmap shows the log2changefold (‘Sweet Jonathan’ vs ’Jonathan’) of the differentially expressed genes.

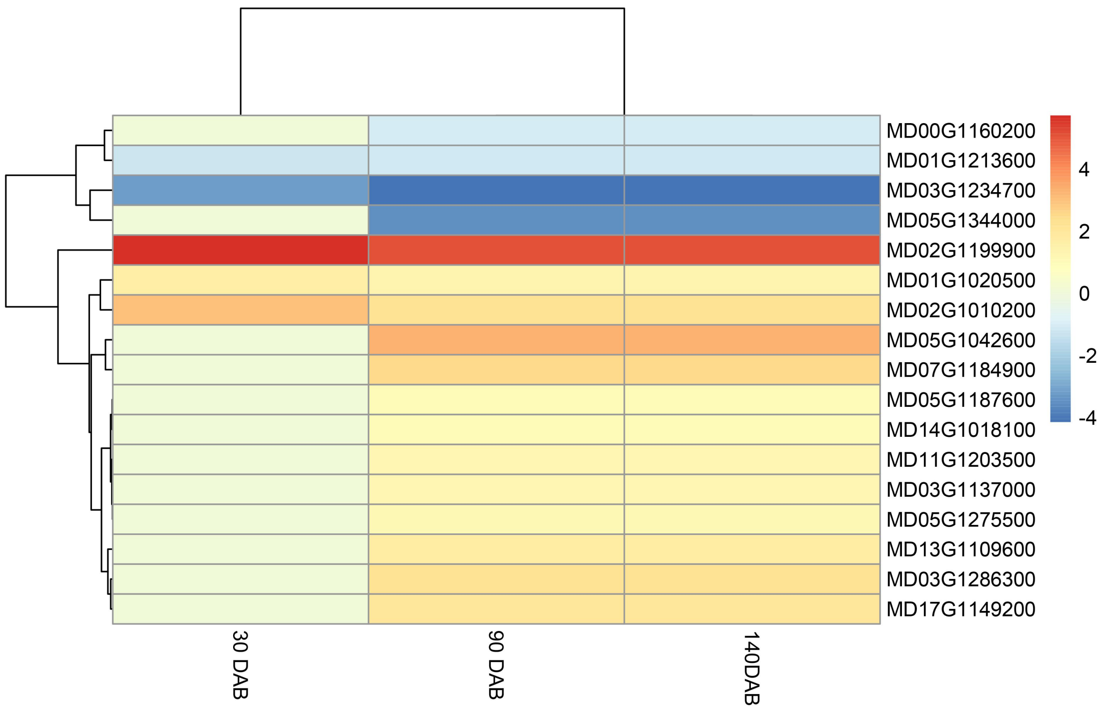

Figure S8

Heatmap shows the log2changefold ('Sweet Jonathan' vs 'Jonathan') of the differentially expressed ATP-binding cassette (ABC) transporter genes.

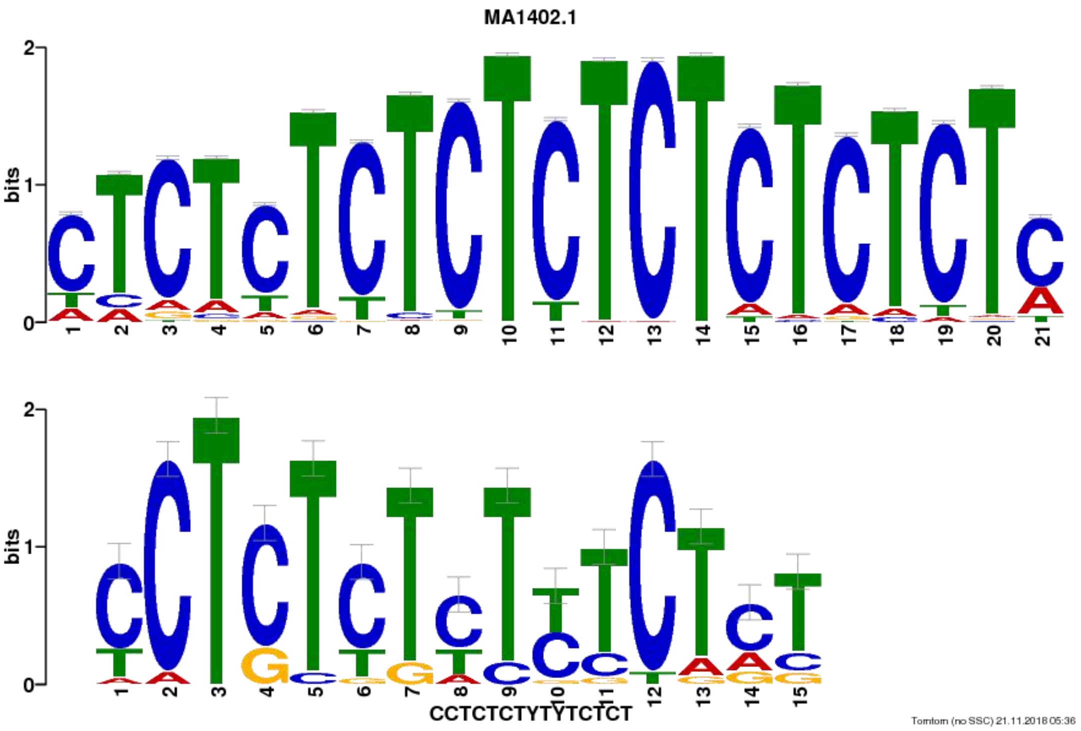

Figure S9a TOMTOM motif alignment result of motif MA1402.1 and the consensus motif, detected in the 17 differentially expressed genes.

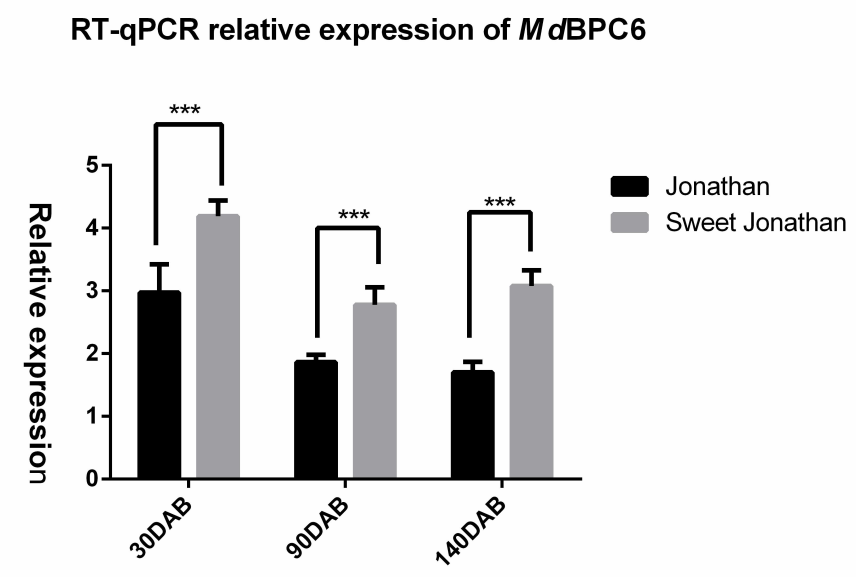

Figure S9b RT-qPCR relative expression of *Md*BPC6

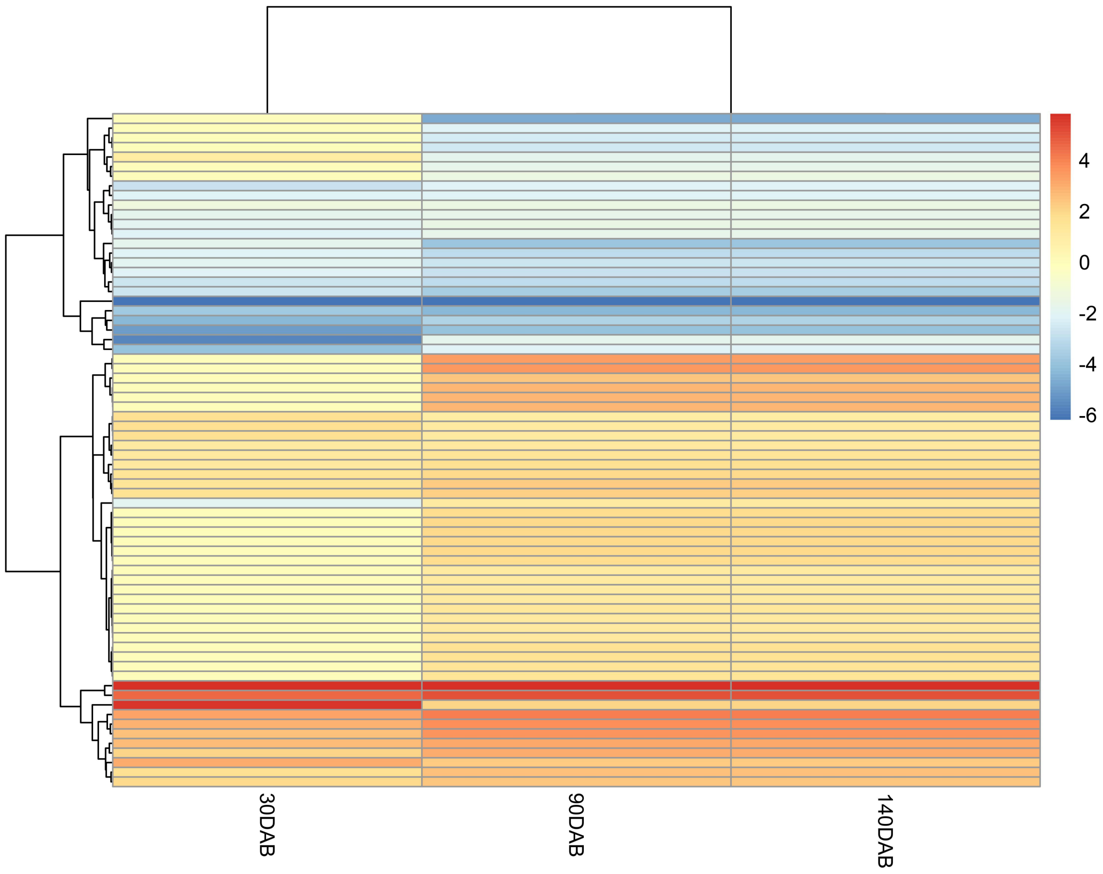

Figure S10 Heatmap shows the log2changefold (‘Sweet Jonathan’ vs ’Jonathan’) of the differentially expressed disease resistant genes
